# ANP32A-mediated histone 3 K27 acetylation is essential for sotorasib activity in KRAS-mutant non–small cell lung cancer

**DOI:** 10.1016/j.jbc.2025.111110

**Published:** 2025-12-29

**Authors:** Kailing Pan, Mingjing Dang, Bo Xu, Zan Huang, Xianguo Chen

**Affiliations:** 1 Department of Cardiothoracic Surgery, Affiliated Jinhua Hospital of Zhejiang University School of Medicine, Jinhua, China; 2 Central Laboratory and Precision Medicine Center, Affiliated Jinhua Hospital of Zhejiang University School of Medicine, Jinhua, China; 3 Jinhua Key Laboratory of Cancer Nutrition and Metabolism Research, Jinhua Municipal Central Hospital, Affiliated Jinhua Hospital of Zhejiang University School of Medicine, Jinhua, China; 4 College of Life Sciences, Hubei Key Laboratory of Cell Homeostasis, Wuhan University, Wuhan, Hubei, China; 5 College of Mathematical Medicine, Zhejiang Normal University, Jinhua, China

**Keywords:** ANP32A, non–small cell lung cancer, sotorasib, YEATS4, H3K27Ac

## Abstract

Drug resistance is a major challenge for the target therapy of *KRAS*-mutant non–small cell lung cancer (NSCLC). Here, we observe that ANP32A is tightly associated with *KRAS*-mutant NSCLC and serves as an unfavorable prognosis factor. ANP32A deficiency impaired cell proliferation, migration, invasion, and cell cycle progression and induced sotorasib (Sot) resistance in *KRAS*-mutant NSCLC cells, which were reversed by ANP32A reoverexpression in ANP32A-deficient cells. Mechanistically, ANP32A deficiency impaired histone 3 acetylation at lysine 27 (H3K27Ac). Particularly, ANP32A deficiency reduced H3K27Ac of the *YEATS4* gene promoter and downregulated YEATS4 expression. ANP32A also interacted with YEATS4 and promoted its binding to H3K27Ac. Furthermore, YEATS4 overexpression partially restored ANP32A deficiency–impaired cell proliferation, H3K27Ac, and Sot sensitivity. Most importantly, trichostatin A mimicked the effect of ANP32A, which restored YEATS4 expression, antagonized Sot resistance, and resensitized ANP32A-deficient cells to Sot *in vitro* and *in vivo*, possibly by reactivating the p53 pathway. Our study identifies a new epigenetic mechanism involving the ANP32A that promotes *KRAS*-mutant lung cancer growth and affects Sot activity. The combination of Sot and histone deacetyltransferase inhibitors could be an effective treatment for *KRAS*-mutant lung cancer. ANP32A may serve as a biomarker for Sot treatment.

*KRAS* mutations are common driver mutations in non–small cell lung cancer (NSCLC), particularly in smokers, and account for about 25% to 30% of NSCLC cases, with *KRAS* G12C being the most frequent mutation (1, 2). Recent advancements in small-molecule inhibitor design have led to the development of targeted therapies aiming at the *KRAS* G12C mutation (3). These inhibitors, such as sotorasib (Sot) and adagrasib, irreversibly bind to the mutant KRAS protein, lock it in an inactive state, disrupt downstream signaling pathways critical for tumor growth and survival, and demonstrate clinical efficacy in treating NSCLC patients with *KRAS* G12C mutation (4). However, limited efficacy alongside toxicity urges exploration of combination strategies to overcome these difficulties. Several combinations are currently under clinical investigation, and promising approaches include combinations of KRAS G12C inhibitors with immune checkpoint inhibitors, SOS1 and SHP2 inhibitors, and platinum-based chemotherapy (3, 5). Tolerability and biomarkers remain critical challenges that must be carefully assessed in these combination therapies. Nevertheless, these studies inspire further investigation into more combination strategies.

To explore potential targets for combination therapy, we noticed that ANP32A, an acidic leucine–rich nuclear phosphoprotein, was an unfavorable prognosis factor, particularly in *KRAS*-mutant lung cancers. ANP32A promoted the proliferation of *KRAS*-mutant lung cancer cells and was required for the sensitivity to Sot treatment. ANP32A is a member of the inhibitor of the histone acetyltransferase complex and preferentially binds to unmodified histone H3 tails *in vitro* (6, 7). Functionally, ANP32A promoted cell proliferation in multiple solid tumors and leukemia (8, 9, 10, 11). Mechanistically, ANP32A knockdown decreased H3 acetylation on interferon-stimulated genes in cervical carcinoma HeLa S3 cells (12). Loss of ANP32A activity reduced levels of histone acetylation in prostatic cancer and leukemia (8, 10). However, mechanisms underlying ANP32A-mediated acetylation have never been clarified. Although a previous study implied a role of ANP32A in apoptosis in lung cancer cells (13), whether and how ANP32A may play a role in *KRAS*-mutant lung cancer has never been addressed.

To explore the underlying mechanisms, ANP32A was shown to enhance histone 3 acetylation at lysine 27 (H3K27Ac) by upregulating and interacting with YEATS4. YEATS4 is known to be a chromatin reader and specifically recognizes and binds H3K14Ac and H3K27Ac on the promoters of actively transcribed genes (14, 15, 16). Specifically, YEATS4 plays a crucial role in regulating chromatin structure and gene transcription by contributing to the assembly of two important multisubunit chromatin remodeling complexes: the SNF2-related CBP activator protein (SRCAP) and the TIP60–p400–TRRAP complex, both of which are involved in the regulation of H2A–H2A.Z exchange and histone acetylation (17). Abnormal expression of the YEATS4 gene is closely related to various diseases, such as cancer and neurodegenerative diseases (17). It is likely that YEATS4 may play important roles mediating the function of ANP32A in histone acetylation and lung cancer.

In this study, we found that ANP32A upregulated the *YEATS4* gene *via* enhancing H3K27Ac in the promoter region. ANP32A also interacted with YEATS4, which enhanced YEATS4 binding to H3K27Ac. Furthermore, YEATS4 restored H3K27Ac, cell proliferation, and Sot sensitivity in ANP32A-deficient cells. Most importantly, histone deacetyltransferase (HDAC) inhibitor trichostatin A (TSA) offset the effect of ANP32A deficiency and potently restored the sensitivity of ANP32A-deficient *KRAS*-mutant lung cancer cells to Sot. TSA potently synergized with Sot and suppressed lung cancer, possibly by reactivating the p53 pathway *in vitro* and *in vivo*. This study reveals novel mechanisms by which ANP32A promotes *KRAS*-mutant lung cancer and its sensitivity to target therapy, in part by upregulating H3K27Ac *via* YEATS4. ANP32A may serve as a biomarker for Sot treatment. The combinatory treatment with Sot and HDAC inhibitors may be a novel strategy to improve the efficacy of target therapy in *KRAS*-mutant lung cancer.

## Results

### ANP32A is an unfavorable prognosis factor in *KRAS*-mutant NSCLC

**Figure 1:**
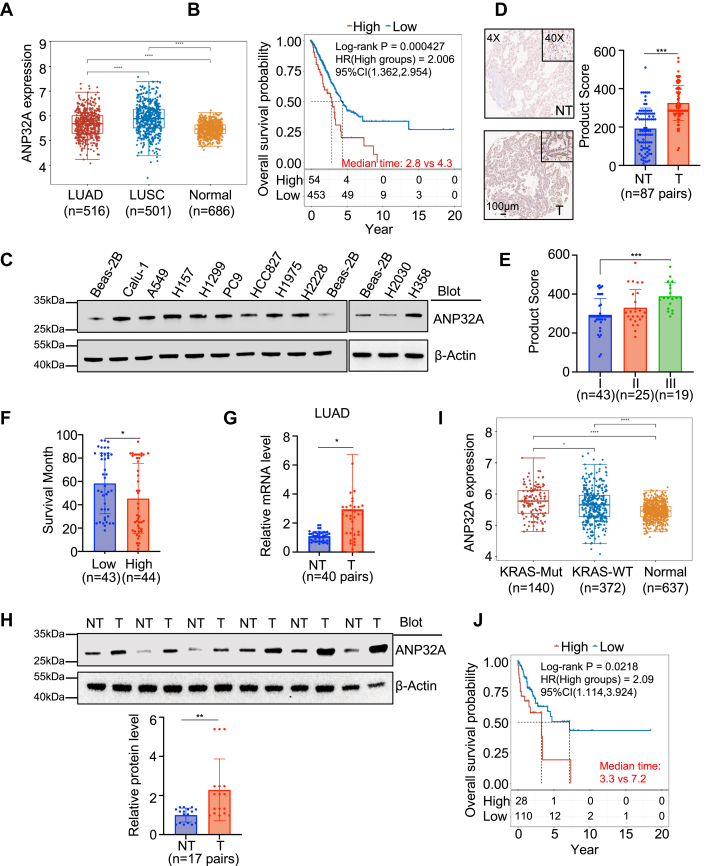
**ANP32A is an unfavorable prognosis factor in *KRAS*-mutant NSCLC.***A*, *ANP32A* gene expression in lung adenocarcinoma (LUAD) and lung squamous cell carcinoma (LUSC) is higher than that in normal tissues in the TCGA data. *B*, Kaplan–Meier survival analysis on TCGA data shows a negative correlation of *ANP32A* gene expression and the overall survival rate in LUAD patients. *C*, Western blot analysis on ANP32A protein expression in nontransformed Beas-2B cells and various LUAD cell lines. *D*, representative images of immunohistochemical staining (*left*) and statistical analysis (*right*) of ANP32A protein in tumor (T) tissues and the paired adjacent nontumor (NT) tissues from 87 LUAD patients (n = 87, *∗∗∗p <* 0.001). *E*, positive correlation of ANP32A protein expression and World Health Organization grade in LUAD patients (n = 43, 25, and 19, *∗∗∗p <* 0.001). *F*, LUAD patients with high ANP32A expression (n = 44) exhibit a longer survival time than LUAD patients with low ANP32A expression (n = 43, *∗p =* 0.035). *G*, LUAD tumor tissues have higher ANP32A expression than the paired adjacent NT tissues (n = 40, *∗p =* 0.028). *H*, Western blot (*top*) and the densitometric analysis (*bottom*) on ANP32A protein expression in LUAD tumor tissues and the paired adjacent NT tissues (n = 17, *∗∗p =* 0.002). *I*, *KRAS*-mutant (KRAS-Mut) LUAD tumor tissues (n = 140) have higher *ANP32A* expression than *KRAS*-wildtype (KRAS-WT) LUAD tumors (n = 372) and normal tissues (normal) (n = 637) in TCGA data. *J*, Kaplan–Meier survival analysis on TCGA data shows a negative correlation of *ANP32A* expression and overall survival rate in *KRAS*-mutant LUAD patients. ∗ indicates *p <* 0.05, ∗∗ indicates *p <* 0.01, and ∗∗∗ indicates *p <* 0.001. NSCLC, non–small cell lung cancer; TCGA, The Cancer Genome Atlas.

We investigated the potential function of ANP32A in lung cancer by analyzing The Cancer Genome Atlas dataset. *ANP32A* was upregulated in both lung adenocarcinoma (LUAD) and lung squamous cell carcinoma (LUSC) (Fig. 1*A*). Interestingly, the expression of *ANP32A* was negatively correlated to the overall survival (OS) rate in LUAD but not in LUSC (Figs. 1*B* and S1*A*). ANP32A protein expression was upregulated in almost all lung cancer cell lines (Figs. 1*C* and S1*B*). Analysis on tissue array confirmed that LUAD tumor tissues (T) expressed higher levels of ANP32A protein than the paired nontumor tissues (Fig. 1*D*). Although the correlation of ANP32A protein expression level and the OS rate was not significant, LUAD patients with high ANP32A protein expression (high) exhibited shorter survival months than LUAD patients with low ANP32A protein expression (low), and ANP32A protein expression was positively correlated to the World Health Organization grade (Figs. 1, *E*, *F*, S1*C*, and Table 1). Upregulation of ANP32A at both mRNA and protein levels was verified in our LUAD but not LUSC patient cohort (Figs. 1, *G*, *H* and S1*D*). Moreover, *ANP32A* upregulation was correlated to *KRAS* mutations (KRAS-Mut) but not other driver mutations, and *ANP32A* expression level was negatively correlated to the OS rate in these *KRAS*-mutant lung cancer patients (Figs. 1, *I*, *J* and S1, *E*–*I*). These findings suggest that ANP32A is an unfavorable prognosis factor in LUAD and may play important roles in *KRAS*-mutant lung cancer.

**Table 1:** The characteristics of LUAD patients

Clinical parameters	Overall, n = 87^a^	ANP322A		Statistics	*p*-value^b^
		Low	High		
		n = 43 (49%)^a^	n = 44 (51%)^a^		
Age	59.51 (9.03)	59.58 (8.22)	59.43 (9.85)	0.08	0.939
Gender				0.01	0.919
Women	41 (47.13%)	21 (48.84%)	20 (45.45%)		
Man	46 (52.87%)	22 (51.16%)	24 (54.555)		
Death				0.09	0.762
Live	30 (34.48%)	16 (37.21%)	14 (31.82%)		
Dead	57 (65.52%)	27 (62.79%)	30 (68.18%)		
Survival month	51.00 [25.00, 82.00]	56.00 [33.00, 83.00]	34.00 [18.75, 80.50]	1215.5	0.022
World Health Organization grade					
I	43 (49.43%)	27 (62.79%)	16 (36.36%)		
II	25 (28.74%)	11 (25.58%)	14 (31.82%)		
III	19 (21.84%)	5 (11.63%)	14 (31.82%)		
T					0.027
T1	49 (56.32%)	30 (69.77%)	19 (43.18%)		

a Mean (SD); n (%); median [IQR].

b Two-sample *t* test; Pearson’s Chi-squared test; Wilcoxon rank-sum test; Fisher’s test for count data with simulated *p* value (based on 2000 replicates).

### ANP32A promotes the proliferation of *KRAS*-mutant lung cancer cells *in vitro*

**Figure 2:**
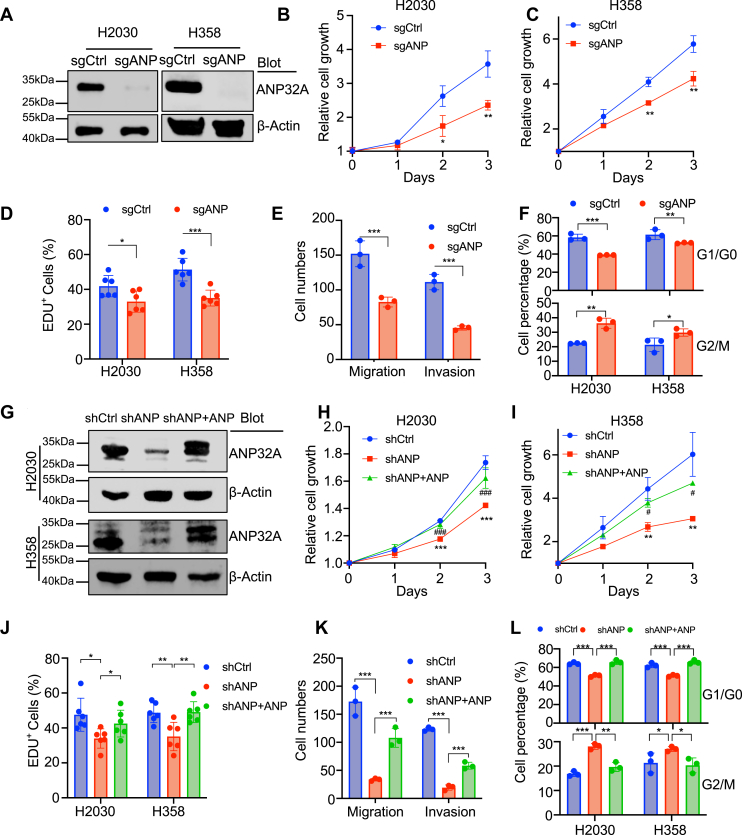
**ANP32A promotes the proliferation of *KRAS*-mutant lung cancer cells.***A*, Western blot analysis on ANP32A protein in control (sgCtrl) and knockout (sgANP) H2030 and H358 cells. *B* and *C*, CCK-8 assay measured cell proliferation in sgCtrl and sgANP H2030 (*B*, *∗p =* 0.024, *∗∗p =* 0.007) and H358 (*C*, *∗∗p =* 0.002 *versus* day 2, 0.006 *versus* day 3) cells as indicated (n = 3). *D*–*F*, statistical analyses of EdU (*D*, *∗p =* 0.03, *∗∗∗p <* 0.001), Transwell (*E*, *∗∗∗p <* 0.001), and cell cycle profile (*F*) in sgCtrl and sg ANP H2030 and H358 cells as indicated (n = 3). Percentage of G1/G0 (*top panel*, *∗∗p =* 0.009, *∗∗∗p <* 0.001) and G2/M (*bottom panel*, *∗p =* 0.024, *∗∗p =* 0.001) was shown in *F*. *G*, Western blot analysis on ANP32A protein in control (shCtrl), knockdown (shANP), and knockdown with ANP32A reintroduction (shANP + ANP) H2030 and H358 cells. *H* and *I*, CCK-8 assay measured cell proliferation in shCtrl, shANP, shANP + ANP H2030 (*H*, *∗∗∗p <* 0.001, *###p <* 0.001) and H358 (*I*, *∗∗p =* 0.002 *versus* day 2, 0.002 *versus* day 3; *#p =* 0.018 *versus* day 2, 0.034 *versus* day 3) cells as indicated (n = 3). *J*–*L*, statistical analyses of EdU (*J*, *∗p =* 0.013 *versus* shCtrl, 0.031 *versus* shANP; ∗∗*p* = 0.008 *versus* shCtrl, 0.007 *versus* shANP), Transwell (*K*, *∗∗∗p <* 0.001), and cell cycle profile (*L*) in shCtrl, shANP, shANP + ANP H2030 and H358 cells as indicated (n = 3). Percentage of G1/G0 (*top panel*, *∗∗∗p <* 0.001) and G2/M (*bottom panel*, *∗p =* 0.029 *versus* shCtrl, 0.01 *versus* shANP, ∗∗*p* = 0.002, ∗∗∗*p* < 0.001) is shown in *L*. ∗ indicates *p <* 0.05, ∗∗ indicates *p <* 0.01, and ∗∗∗ indicates *p <* 0.001; # indicates *p <* 0.05, ## indicates *p <* 0.01, and ### indicates *p <* 0.001 compared with the shANP group in (*H*) and (*I*). CCK-8, Cell Counting Kit-8; Edu, 5-ethynyl-2′-deoxyuridine.

The function of ANP32A in lung cancer was tested by knocking out ANP32A in H2030 and H358 cells (Figs. 2*A* and S2, *A*–*B*). Apparently, ANP32A knockout cells (sgANP) exhibited impaired cell proliferation, colony formation, migration and invasion, and arrested the cell cycle at G2/M phase compared with control cells (sgCtrl) (Figs. 2, *B*–*F* and S2, *C*–*F*). To verify the specific effect of ANP32A, ANP32A knockdown cells (shANP) and ANP32A knockdown with ANP32A reoverexpression cells (shANP + ANP) were established (Figs. 2*G* and S2*G*). As expected, shANP cells showed similar phenotypes to sgANP cells, and these phenotypes were reversed in shANP + ANP cells (Figs. 2, *H*–*L* and S2, *H*–*J*). In addition, ANP32A deficiency did not significantly induce apoptosis (Fig. S2, *K* and *L*). These results suggest that ANP32A upregulation promotes the proliferation, migration, and invasion of lung cancer cells *in vitro*.

### ANP32A is essential for the sensitivity of *KRAS*-mutant cells to Sot

**Figure 3:**
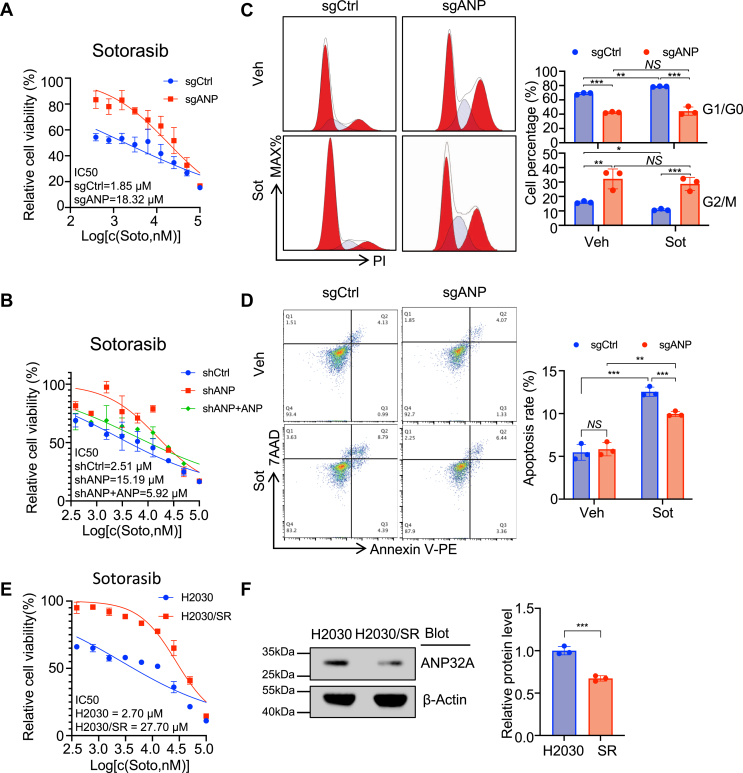
**ANP32A is essential for the sensitivity of *KRAS*-mutant cells to sotorasib (Sot).***A*, the CCK-8 assay measured the IC_50_ of control (sgCtrl) and ANP32A-knockout (sgANP) H2030 cells subjected to Sot treatment (n = 3). *B*, the CCK-8 assay measured the IC_50_ of shCtrl, shANP, and shANP + ANP H2030 cells subjected to Sot treatment (n = 3). *C*, representative flow cytometry (*left*) and statistical analysis (*right*) of cell cycle profile in sgCtrl and sgANP H2030 cells (n = 3) treated with control (vehicle; Veh) or Sot (1 μM). Percentage of G1/G0 (*top panel*, *∗∗p =* 0.004, ∗∗∗*p* < 0.001) and G2/M (*bottom panel*, *∗p =* 0.041, ∗∗*p* = 0.001, and ∗∗∗*p* < 0.001) is shown. *D*, representative flow cytometry (*left*) and statistical analysis (*right*, ∗*∗p =* 0.002, ∗∗∗*p* < 0.001) of cell apoptosis in sgCtrl and sgANP H2030 cells treated with control (Veh) or Sot (1 μM) (n = 3). *E*, the CCK-8 measured the IC_50_ of Sot in the parental H2030 cells and Sot-resistant cells (H2030/SR) (n = 3). *F*, Western blot analysis on ANP32A protein in H2030 and H2030/SR cells (n = 3, ∗∗∗*p <* 0.001). ∗ indicates *p <* 0.05, ∗∗ indicates *p <* 0.01, and ∗∗∗ indicates *p <* 0.001. CCK-8, Cell Counting Kit-8; NS, nonsignificance.

Given that *KRAS*-mutant lung cancer expressed higher levels of *ANP32A* than wildtype lung cancer, we further tested whether ANP32A may play a role in target therapy of *KRAS*-mutant lung cancer. Notably, ANP32A knockout (sgANP) and knockdown (shANP) H2030 cells showed much higher IC_50_ of Sot than control cells (sgCrtl and shCtrl), and reoverexpression of ANP32A in shANP cells reduced the IC_50_ (Fig. 3, *A* and *B*). Although Sot treatment (1 μM) significantly caused cell cycle arrest at G1/G0 phase in sgCtrl H2030 cells, it barely affected cell cycle profile in sgANP H2030 cells (Fig. 3*C*). Moreover, sgANP cells exhibited less Sot-induced apoptosis than sgCtrl cells (Fig. 3*D*). Interestingly, Sot-resistant H2030 cells (H2030/SR) showed ANP32A downregulation compared with the parental H2030 cells (Fig. 3, *E* and *F*). These observations demonstrate that ANP32A may play an essential role in the sensitivity of *KRAS*-mutant cells to Sot.

### ANP32A enhances H3K27 acetylation and interacts with YEATS4

**Figure 4:**
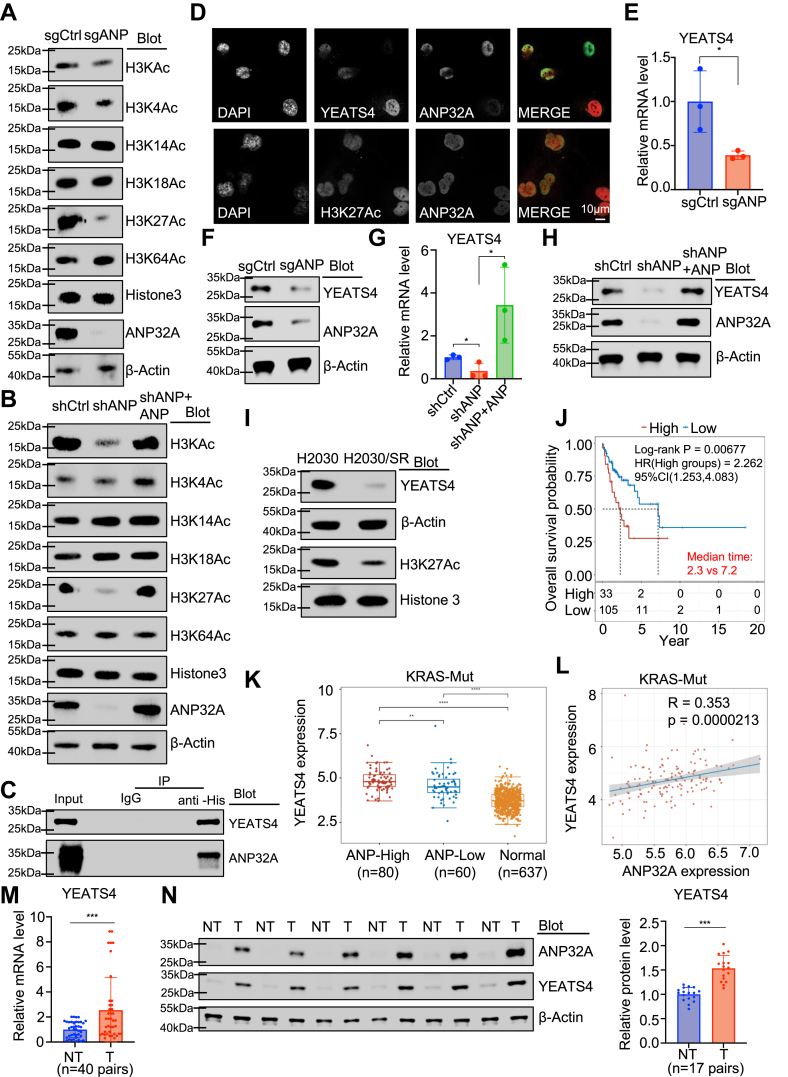
**ANP32A regulates H3K27 acetylation and interacts with YEATS4.***A* and *B*, Western blot showed different histone H3 acetylation levels at various sites in sgCtrl and sgANP H2030 cells (*A*) or in shCtrl, shANP, and shANP + ANP H2030 cells (*B*). *C*, coimmunoprecipitation with anti-His antibody, followed by Western blot using His-ANP32A–overexpressing 293T cells was performed to confirm ANP32A interaction with YEATS4. *D*, immunofluorescence showed ANP32A colocalization with YEATS4 (*top*) and H3K27Ac (*bottom*). *E* and *F*, quantitative RT–PCR (*E*, n = 3, *∗p =* 0.04) and Western blot (*F*) analyses on YEATS4 mRNA and protein levels in sgCtrl and sgANP H2030 cells (n = 3). *G* and *H*, quantitative RT–PCR (*G*, n = 3, *∗p =* 0.02 *versus* shCtrl, 0.02 *versus* shANP) and Western blot (*H*) analyses evaluated YEATS4 mRNA and protein levels in shCtrl, shANP, and shANP + ANP H2030 cells (n = 3). *I*, Western blot analysis assessed YEATS4 and H3K27Ac levels in H2030 and H2030/SR cells. *J*, Kaplan–Meier survival analysis on TCGA data shows a negative correlation of *YEATS4* expression and overall survival rate in *KRAS*-mutant LUAD patients. *K*, *ANP32A*-high expression (KRAS-high) LUAD tumor tissues (n = 80) have higher *YEATS4* expression than *ANP32A*-low expression (KRAS-low) LUAD tumors (n = 60) and normal tissues (normal) (n = 637) in TCGA data. *L*, *ANP32A* expression was positively correlated with *YEATS4* in *KRAS*-mutant patients. *M*, LUAD tumor (T) tissues have higher *YEATS4* mRNA level than the paired adjacent nontumor (NT) tissues (n = 40, *∗∗∗p <* 0.001). *N*, Western blot (*left*) and the densitometric analysis (*right*) on YEATS4 and ANP32A protein expression in LUAD tumor (T) tissues and the paired adjacent NT tissues (n = 17, *∗∗∗p <* 0.001). ∗ indicates *p <* 0.05, ∗∗ indicates *p <* 0.01, and ∗∗∗ indicates *p <* 0.001. H3K27Ac, histone 3 acetylation at lysine 27; LUAD, lung adenocarcinoma; TCGA, The Cancer Genome Atlas.

ANP32A has been shown to regulate H3 acetylation (10). Indeed, sgANP cells displayed reduced histone H3 acetylation, particularly at lysine 4 and 27 sites (H3K4Ac and H3K27Ac) (Figs. 4*A* and S3*A*). The reduced H3K27Ac was confirmed in shANP H2030 cells, and ANP32A reoverexpression in these cells (shANP + ANP) reversed the effect, whereas ANP32A knockdown only mildly affected H3K4Ac (Figs. 4*B* and S3*B*). Since ANP32A is not an acetyltransferase, ANP32A may mediate H3K27Ac by recruiting partners. Analysis on potential ANP32A-interacting proteins (biogrid.org) suggests that BRD4 and YEATS4 involving histone acetylation may interact with ANP32A. FLAG-YEATS4 but not FLAG-BRD4 was coimmunoprecipitated with His-ANP32A (Figs. 4*C* and S3*C*). Immunofluorescent experiment demonstrated that merging of ANP32A and YEATS4 or H3K27Ac gave a yellow signal, confirming ANP32A colocalization with YEATS4 and H3K27Ac (Fig. 4*D*). ANP32A deficiency downregulated YEATS4 rather than BRD4 at protein and mRNA levels, which was rescued by reintroduction of ANP32A (Figs. 4, *E*–*H* and S3, *D*–*G*). MG132 treatment did not normalize YEATS4 expression in these cells, excluding possible involvement of the proteosome pathway (Fig. S3*H*). Moreover, H2030/SR cells exhibited downregulation of YEATS4 and H3K27Ac compared with the parental H2030 cells (Figs. 4*I* and S3*I*). In support, we found that *YEATS4* served as a prognostic factor in KRAS-mutant NSCLC (Fig. 4*J*). Moreover, *YEATS4* expression levels in the high *ANP32A* expression group (ANP-high) were higher than those in the low ANP32A expression group (ANP-low), and *YEATS4* expression was positively correlated with *ANP32A* expression in *KRAS*-mutant NSCLC (Fig. 4, *K* and *L*). Consistent with the findings for ANP32A, upregulation of YEATS4 at both the mRNA and protein levels was confirmed in our LUAD patient cohort (Fig. 4, *M* and *N*). Considering the function of YEATS4 in assembling SRCAP and TIP60–p400–TRRAP complexes for histone acetylation, ANP32A may promote Sot sensitivity by enhancing H3K27Ac *via* upregulating and interacting with YEATS4.

### ANP32A promotes H3K27Ac by upregulating and interacting with YEATS4

**Figure 5:**
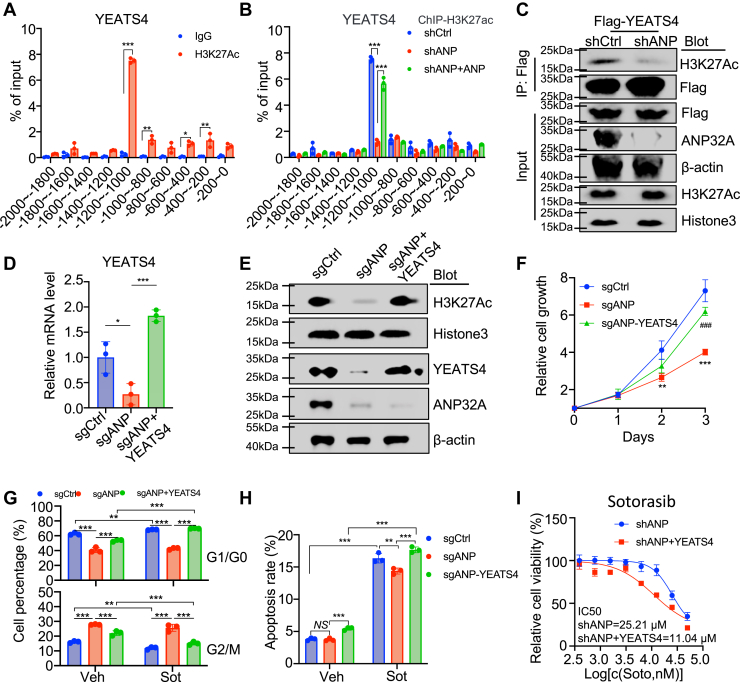
**ANP32A promotes H3K27Ac by upregulating and interacting with YEATS4.***A*, ChIP–qPCR measured H3K27Ac enrichment at the different regions of *YEATS4* gene promoter in H2030 cells (n = 3, ∗*p =* 0.011, ∗*p =* 0.002, and *∗∗∗p <* 0.001). *B*, ChIP–qPCR evaluated H3K27Ac enrichment at the different regions of *YEATS4* gene promoter in shCtrl, shANP, and shANP + ANP H2030 cells (n = 3, ∗*∗∗p <* 0.001). *C*, FLAG-YEATS4 was overexpressed in shCtrl or shANP 293T cells. Cell lysates were used for coimmunoprecipitation with an anti-FLAG antibody followed by Western blot to confirm that ANP32A knockdown impaired the interaction of YEATS4 and H3K27Ac. *D* and *E*, qRT–PCR measured *YEATS4* expression (*D*, n = 3, ∗*p =* 0.02, ∗*∗∗p <* 0.001) and Western blot analyzed ANP32A, YEATS4, and H3K27Ac levels (*E*) in sgCtrl, sgANP, and sgANP + YEATS4 H2030 cells. *F*, CCK-8 assays measured cell proliferation in shCtrl, shANP, and shANP + YEATS4 H2030 cells (n = 3 ∗∗*p =* 0.009, *∗∗∗p <* 0.001, and *###p <* 0.001). *G*–H, flow cytometry analyzed on cell cycle (*G*) and apoptosis (*H*, ∗*∗p =* 0.002, *∗∗∗p <* 0.001) in shCtrl, shANP, and shANP + YEATS4 H2030 cells treated with control (vehicle [Veh]) or sotorasib (Sot, 1 μM) (n = 3). Percentage of G1/G0 (*top panel*, ∗*∗p =* 0.002, ∗*∗∗p <* 0.001) and G2/M (*bottom panel*, *∗∗p =* 0.002, ∗*∗∗p <* 0.001) is shown in *G*. *I*, CCK-8 assay determined IC_50_ of Sot in shANP and shANP + YEATS4 H2030 cells (n = 3). ∗ indicates *p <* 0.05, ∗∗ indicates *p <* 0.01, and ∗∗∗ indicates *p <* 0.001. # indicates *p <* 0.05, ## indicates *p <* 0.01, and ### indicates *p <* 0.001 compared with the sgANP group in (*F*). CCK-8, Cell Counting Kit-8; ChIP–qPCR, chromatin immunoprecipitation–quantitative PCR; H3K27Ac, histone 3 acetylation at lysine 27; NS, nonsignificance.

We further tested whether H3K27Ac mediated ANP32A function. Chromatin immunoprecipitation (ChIP)–quantitative PCR (qPCR) revealed a significant enrichment of H3K27Ac on the upstream of *YEATS4* gene and YEATS4 target gene *LMO4* gene promoters, particularly *YEATS4* gene in the region between −1200 bp and −1000 bp and *LMO4* gene in the region between −800 bp and −200 bp (Figs. 5*A* and S4*A*). Furthermore, H3K27Ac in these regions was impaired in shANP cells but restored in shANP + ANP cells (Figs. 5*B* and S4*B*). In shANP cells, much less of H3K27Ac was coimmunoprecipitated with FLAG-YEATS4 than that in shCtrl cells, suggesting that ANP32A deficiency impaired the interaction of YEATS4 with H3K27Ac (Figs. 5*C* and S4*C*). Restoration of *YEATS4* expression in ANP32A knockout cells (sgANP + YEATS4) did not affect *ANP32A* expression but partially rescued the *LMO4* expression (Figs. 5, *D*, *E* and S4, *D*, *E*). Notably, YEATS4 overexpression in ANP32A-knockout cells (sgANP + YEATS4) restored ANP32A knockout–impaired H3K27Ac, cell proliferation, and cell cycle profile (Figs. 5, *E*–*G* and S4, *F*, *G*). YEATS4 also potently antagonized Sot-induced cell cycle arrest and apoptosis (Figs. 5, *G*, *H* and S4, *G*, *H*). Most importantly, shANP + YEATS4 cells exhibited less IC_50_ of Sot by twofolds than shANP cells (Fig. 5*I*). These results suggest that ANP32A promotes the proliferation and Sot sensitivity of lung cancer cells by enhancing H3K27Ac to upregulate YEATS4 and interacting with YEATS4.

### Inhibition of histone deacetylation enhances Sot efficacy on *KRAS*-mutant lung cancer *in vitro* and *in vivo*

**Figure 6:**
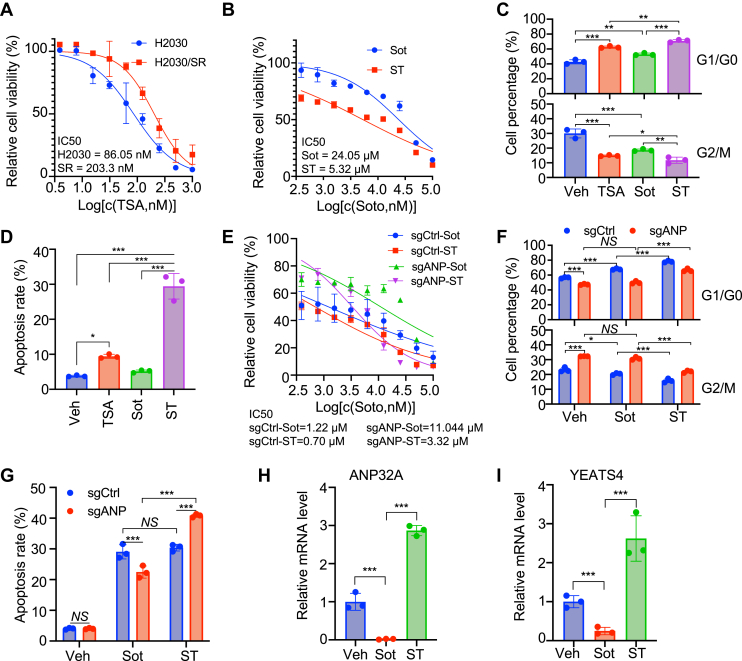
**Trichostatin A (TSA) enhances sotorasib (Sot) efficacy on *KRAS*-mutant lung cancer *in vitro*.***A*, CCK-8 measured the IC_50_ of TSA in H2030 and H2030/SR cells (n = 3). *B*, CCK-8 measured the IC_50_ of Sot alone and in the presence of TSA (100 nM) in H2030/SR cells (n = 3). *C* and *D*, flow cytometry analyzed the cell cycle profile (*C*) and apoptosis (*D*, *∗p =* 0.025, ∗∗*∗p <* 0.001) in H2030/SR cells treated with control (vehicle [Veh]), TSA (100 nM), Sot (1 μM), or their combination (ST) (n = 3). Percentage of G1/G0 (*top panel*, *∗∗p =* 0.001 *versus* Veh, 0.004 *versus* TSA, ∗∗∗*p* < 0.001) and G2/M (*bottom panel*, *∗p =* 0.045, ∗∗*p* = 0.009, and ∗∗∗*p* < 0.001) is shown in *C*. *E*, CCK-8 measured the IC_50_ of Sot alone and in the presence of TSA (100 nM) in sgCtrl and sgANP H2030 cells (n = 3). *F* and *G*, flow cytometry detected the cell cycle profile (*F*, *∗p =* 0.016, ∗∗∗*p* < 0.001) and apoptosis (*G*, *∗∗∗p <* 0.001) in sgCtrl and sgANP H2030 cells treated with Veh, Sot (1 μM), or ST (Sot, 1 μM; TSA, 50 nM) (n = 3). Percentage of G1/G0 (*top panel*) and G2/M (*bottom panel*) is shown in *F*. *H* and *I*, quantitative RT–PCR measured *ANP32A* (*H*, ∗∗∗*p <* 0.001) and *YEATS4* (*I*, ∗∗∗*p <* 0.001) in H2030 cells treated with Veh, Sot (1 μM), or ST (Sot, 1 μM; TSA, 50 nM) (n = 3). ∗ indicates *p < 0*.05, ∗∗ indicates *p <* 0.01, and ∗∗∗ indicates *p <* 0.001. CCK-8, Cell Counting Kit-8; NS, nonsignificance.

**Figure 7:**
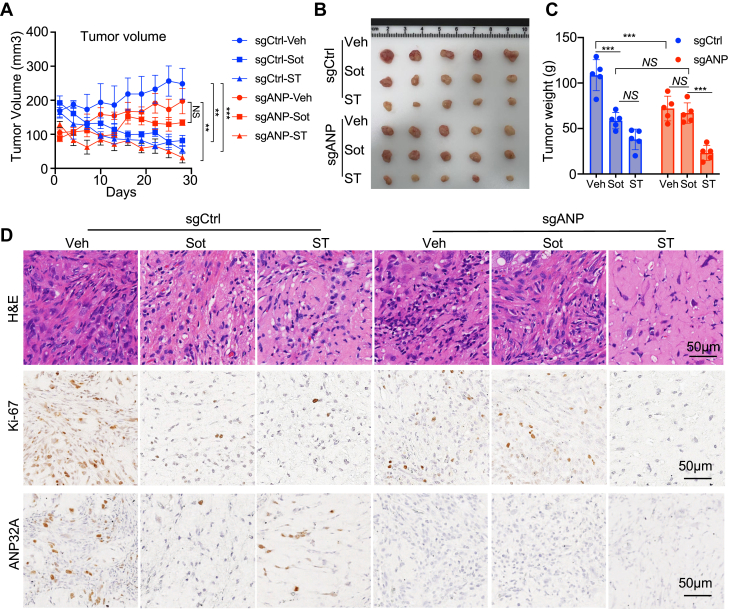
**Trichostatin A (TSA) enhances sotorasib (Sot) efficacy on *KRAS*-mutant lung cancer *in vivo*.***A*, sgCtrl and sgANP H2030 cells were used for xenograft experiments. Tumor-bearing mice were treated with vehicle (Veh), Sot (30 mg/kg), or ST (Sot plus TSA; Sot, 30 mg/kg; TSA, 0.5 mg/kg) for 28 days. Tumor volume was recorded every 3 days (n = 5, *∗∗p =* 0.002 *versus* sgCtrl-Veh, 0.002 *versus* sgANP-Veh, ∗∗∗*p* < 0.001). *B* and *C*, on day 28, mice were euthanized, and tumors were removed (*B*) and weighed (*C*) (n = 5, *∗∗∗p <* 0.001). *D*, images of H&E, Ki-67, and ANP32A staining in tumor sections are shown (n = 5). ∗ indicates *p <* 0.05, ∗∗ indicates *p <* 0.01, and ∗∗∗ indicates *p <* 0.001. NS, nonsignificance.

The promoting effect of ANP32A on H3K27Ac and Sot sensitivity in *KRAS*-mutant lung cancer cells implied that histone acetylation might improve Sot activity. To test this possibility, TSA, as an inhibitor of HDAC, was used to mimic the effect of ANP32A on histone acetylation and treat Sot-resistant H2030 cells. HDAC inhibitors enhance histone acetylation and have been widely used for cancer treatment. H2030/SR cells were relatively resistant to HDAC inhibitor TSA with an IC_50_ of 203.3 nM compared with parental H2030 cells (Fig. 6*A*). The presence of TSA (100 nM) decreased IC_50_ of Sot to less than one-fourth in H2030/SR cells, suggesting that TSA treatment reduced Sot resistance (Fig. 6*B*). Furthermore, TSA (100 nM) or Sot (Sot, 1 μM) alone caused cell cycle arrest at G1/G0 phase and mildly affected apoptosis in H2030/SR cells, whereas the combination of Sot and TSA (ST) significantly worsened phenotypes (Figs. 6, *C*, *D* and S5, *A*, *B*). TSA also potently improved the sensitivity of sgANP cells to Sot (Fig. 6*E*). Sot promoted cell cycle arrest at G1/G0 phase and apoptosis in sgCtrl cells but was less effective in sgANP cells (Figs. 6, *F*, *G* and S5, *C*, *D*). Coadministration of TSA (ST) enhanced Sot-induced cell cycle arrest at G1/G0 phase in both sgCtrl and sgANP cells, whereas TSA only increased Sot-induced apoptosis in sgANP cells but not sgCtrl cells (Figs. 6, *F*, *G* and S5, *C*, *D*). Interestingly, Sot treatment alone decreased the expression of *ANP32A* and *YEATS4*, whereas coadministration of TSA (ST) restored the expression of both genes in H2030 cells (Fig. 6, *H* and *I*). *In vivo*, ANP32A knockout delayed H2030 cells to form subcutaneous tumors and suppressed cell proliferation as expected (Fig. 7, *A*–*D*). Sot treatment alone significantly suppressed tumor formation and cell proliferation in sgCtrl cells but failed to do so in sgANP cells (Fig. 7, *A*–*D*). Most importantly, ST further repressed tumor formation and cell proliferation in sgANP cells, which was more effective than that in sgCtrl cells (Fig. 7, *A*–*D*). These results demonstrate that inhibition of histone deacetylation by TSA enhances the sensitivity of *KRAS*-mutant lung cancer cells to Sot *in vitro* and *in vivo*. TSA treatment partially mimics the effect of ANP32A and promotes Sot efficacy.

### Activation of the p53 signaling pathway *via* ANP32A-mediated histone acetylation

**Figure 8:**
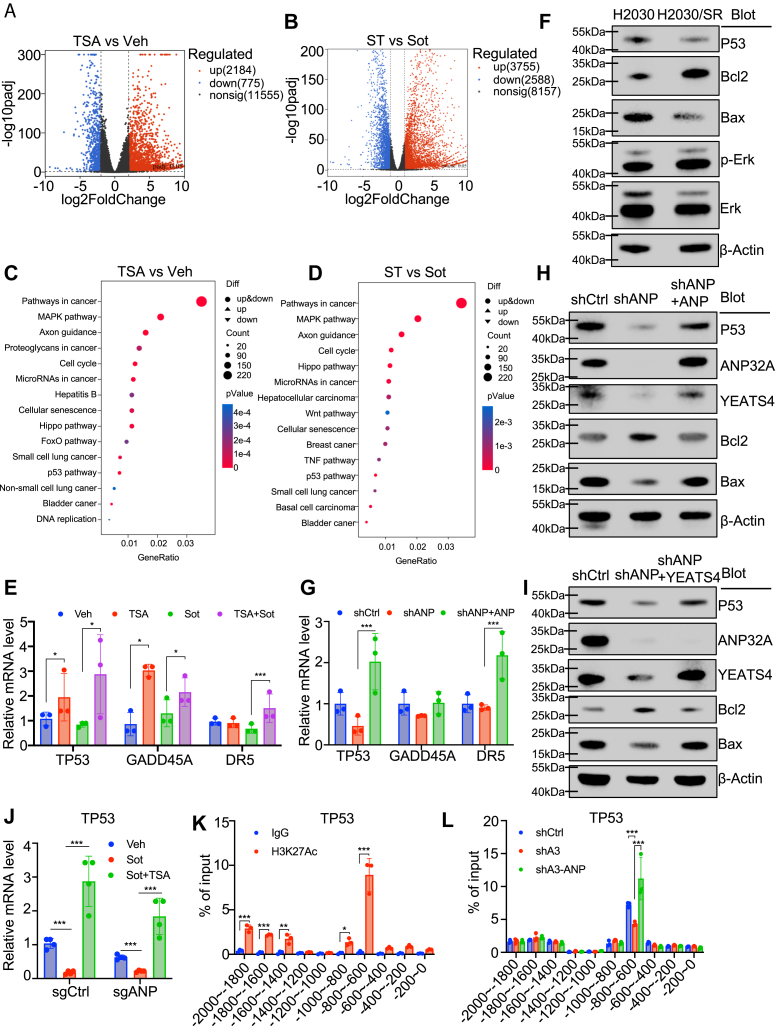
**ANP32A facilitates resistance to sotorasib (Sot) by modulating the p53 signaling pathway *via* histone acetylation.***A* and *B*, Volcano plots show differentially expressed genes in H2030/SR cells treated with vehicle (Veh), Sot (1 μM), TSA (100 nM), or ST for RNA-Seq analysis. TSA-treated group is compared with Veh-treated group in (*A*), and TSA-treated group is compared with Sot-treated group in (*B*). *C* and *D*, KEGG pathway enrichment analyses on the differentially expressed genes identified in *A* and *B* are shown in (*C*) and (*D*), respectively. *E*, the gene expression levels of *TP53*, *GADD45A*, and *DR5* were evaluated in H2030/SR cells after treatment with Veh, TSA (100 nM), Sot (1 μM), or ST (n = 3, *∗*P-TP53 = 0.048 *versus* Veh, 0.016 *versus* Sot, ∗P-GADD45A = 0.011 *versus* Veh, 0.018 *versus* Sot, ∗∗∗*p* < 0.001). *F*, Western blot analysis assessed p53 and mitogen-activated protein kinase signaling molecules in H2030 and H2030/SR cells. *G* and *H*, p53 pathway–related molecule expression was assessed at mRNA (*G*) and protein (*H*) levels in shCtrl, shANP, and shANP + ANP cells (n = 3, *∗∗∗p <* 0.001). *I*, p53 pathway–related molecule expression was assessed at protein levels in shCtrl, shANP, and shANP + YEATS4 cells (n = 3). *J*, *TP53* gene expression was measured in tumor tissues from the mouse model (n = 5, *∗∗∗p <* 0.001). *K*, ChIP–qPCR measured H3K27Ac at the *TP53* gene promoter in H2030 cells (n = 3, *∗p =* 0.018, ∗*∗p =* 0.002, and *∗∗∗p <* 0.001). *L*, ChIP–qPCR evaluated H3K27Ac enrichment at the *TP53* gene promoter regions in shCtrl, shANP, and shANP + ANP H2030 cells (n = 3, ∗∗∗*p <* 0.001). ∗ indicates *p <* 0.05, ∗∗ indicates *p <* 0.01, and ∗∗∗ indicates *p <* 0.001. ChIP–qPCR, chromatin immunoprecipitation–quantitative PCR; H3K27Ac, histone 3 acetylation at lysine 27; KEGG, Kyoto Encyclopedia of Genes and Genomes; ST, combination of Sot and TSA; TSA, trichostatin A.

To dissect how TSA may increase Sot sensitivity, H2030/SR cells were treated with TSA, Sot, or combination (ST), and used for RNA-Seq. As expected, few genes were altered by Sot treatment, whereas amounts of genes were altered by TSA alone or ST (Figs. 8, *A*, *B* and S6, *A*, *B*). Of note, mitogen-activated protein kinase and p53 pathways were among the top-ranked signatures commonly enriched in Kyoto Encyclopedia of Genes and Genomes analysis (Fig. 8, *C* and *D*). p53 and its multiple p53 target genes were activated by TSA (Fig. 8*E*). In support, downregulation of p53 and Bax and upregulation of Bcl2 were observed in H2030/SR cells, whereas the Erk phosphorylation was unchanged (Figs. 8*F* and S6*C*). Moreover, p53 and its target genes were reduced in ANP32A-deficient cells, which were reversed by reintroduction of ANP32A or YEATS4 (Figs. 8, *G*–*I* and S6, *D*–*F*). Notably, Sot treatment downregulated p53, whereas ST reversed the effect of Sot (Figs. 8*J* and S6, *G*–*H*). ChIP–qPCR verified that ANP32A knockdown impaired H3K27Ac in the promoter region of TP53 (Fig. 8, *K* and *L*). Taken together, these observations suggest that activation of the p53 pathway may play an important role in ANP32A-mediated Sot activity in *KRAS*-mutant lung cancer cells.

## Discussion

KRAS G12C–selective inhibitors mark a significant milestone in cancer drug discovery and greatly improve the treatment of lung cancer (18, 19). However, acquired resistance is observed in most patients who initially respond to KRAS G12C inhibition but then develop resistance after 4 to 6 months (20). Despite the eventually developed resistance, multiple combinatorial treatments have been proposed to enhance the efficacy of KRAS G12C inhibitors, including genetic mechanisms and adaptive nongenetic mechanisms (5). There is no doubt that biomarkers are essential for the selection of combinatorial treatments to overcome KRAS G12C inhibitor resistance.

Our study demonstrates that ANP32A is an unfavorable prognosis factor for *KRAS*-mutant lung cancer. ANP32A promoted *KRAS*-mutant lung cancer but caused *KRAS*-mutant lung cancer cells to be sensitive to Sot *in vitro* and *in vivo*. These seemingly controversial observations may reflect the inseparable duality of uncontrolled proliferation in cancer. It not only confers cancer cells of growth advantage but also causes multiple stresses, such as metabolic and replicative stress, redox stress, and genomic instability, and so on, which are potential vulnerable points for cancer therapy. Cancer cells may maintain the balance to favor tumor development, whereas drug intervention may break the balance and push it to the opposite. In our study, ANP32A expression not only promoted cell proliferation but also caused upregulation of apoptosis genes but downregulation of survival genes, which were elegantly balanced. Moreover, Sot treatment or Sot resistance potently suppressed ANP32A and tumor suppressor p53 but upregulated the expression of survival gene, which explained the rapid development of drug resistance. However, TSA treatment reversed p53 expression because of ANP32A deficiency or Sot treatment through regulating H3K27Ac, which increased apoptosis, promoting Sot activity. We also noticed and confirmed that TSA treatment also upregulated p63 and p73 (Fig. S6, *F*–*H*). Thus, we speculate that the ability of ANP32A to activate these apoptosis pathways may determine its role in promoting Sot activity. ANP32A possibly serves as a biomarker for early selection of combinatorial treatment to overcome drug resistance.

Histone acetylation appears to be important for ANP32A-mediated Sot sensitivity. ANP32A has been suggested to enhance or reduce histone acetylation dependent on partners (10). However, the underlying mechanisms have never been addressed. In this study, we demonstrated that YEATS4 was an ANP32A partner that enhanced H3K27Ac. ANP32A upregulated YEATS4 by enhancing H3K27Ac in the *YEATS4* gene promoter region. ANP32A also interacted with YEATS4 and increased YEATS4 interaction with H3K27Ac. Given that ANP32A preferentially binds to unmodified H3 and YEATS4 binds to H3K27Ac, ANP32A likely binds to unmodified H3 and promotes H3K27Ac by recruiting YEATS4, which is a component of SRCAP and the TIP60–p400–TRRAP complexes promoting histone acetylation (17). In turn, H3K27Ac further promotes YEATS4 expression forming a positive feedback loop. This self-reinforcing cycle may ensure the stability of the ANP32A–YEATS4–H3K27Ac axis. ANP32A suppression disrupts this loop contributing to the acquisition of Sot resistance. It should be noted that YEATS4 also functions through other mechanisms such as LncAKHE (21), which is independent of ANP32A. We could not exclude that other factors may also mediate the effect of ANP32A.

Multiple genomic mechanisms and adaptive nongenomic mechanisms underlying drug resistance to KRAS G12C–selective inhibitors have been dissected (22). In our study, we found that the p53 pathway was notably suppressed in Sot-resistant cells and downregulated by ANP32A deficiency and Sot treatment in H2030 cells. ANP32A knockdown and Sot treatment also suppressed the expression of apoptosis gene but upregulated survival protein Bcl2. These observations may explain why ANP32A deficiency causes significant cell cycle arrest but mild apoptosis. Moreover, TSA treatment reactivated Sot-suppressed p53 expression in H2030 cells, which was similar to p53 reactivation by re-expression of ANP32A in shANP cells. It appears that both TSA treatment and ANP32A target the p53 pathway to promote Sot activity. This may explain why TSA treatment partially mimics the effect of ANP32A promoting Sot efficacy. Previous studies showed that TSA promoted p53 acetylation at K382 and activated the p53–Bax apoptotic pathway (23). TSA also suppressed tumors through distinct mechanisms: inducing p21-mediated G1/S arrest in wildtype p53 cells or triggering mitotic catastrophe in p53-mutant cells (24). Our findings are consistent to these observations and suggest that the p53 pathway may play a critical role in Sot resistance. Reactivation of p53 pathway may be an alternative strategy to improve Sot therapy.

Our study also proposes novel strategies for combinatory treatment. Previously, we showed that direct targeting of ANP32A with a small peptide by blocking ANP32A binding to H3 potently repressed leukemia (25). Considering the effect of ANP32A in *KRAS*-mutant lung cancer, such a peptide is likely to suppress lung cancer and enhance Sot efficacy. Moreover, we showed that TSA potently reversed the effect of ANP32A deficiency and potently sensitized *KRAS*-mutant lung cancer cells to Sot *in vitro* and *in vivo*. Since HDAC inhibitors have been developed and widely used in clinics (26), it is worthy of testing the combinatorial treatment with KRAS G12C–selective inhibitors and HDAC inhibitors.

In summary, mutant KRAS signaling activates the ANP32A–YEATS4–H3K27Ac axis to retain the expression of apoptosis genes in lung cancer. Mutant KRAS signaling also promotes survival and proliferation. Without drug intervention, it is well balanced to favor proliferation and retain the drug sensitivity. Sot treatment suppresses proliferation and impairs the ANP32A–YEATS4–H3K27Ac axis, which causes reduced proliferation and eventually develops drug resistance. TSA coadministration restores ANP32A–YEATS4–H3K27Ac axis and activates apoptosis pathway to reverse drug resistance (Fig. S6*I*).

## Experimental procedures

### Cell culture

Cell lines Beas-2B (Research Resource Identifier [RRID]: CVCL_0168), Calu-1 (RRID: CVCL_0608), A549 (RRID: CVCL_0023), H157 (RRID: CVCL_0463), H1299 (RRID: CVCL_0060), PC9 (RRID: CVCL_B260), HCC827 (RRID: CVCL_2063), H1975 (RRID: CVCL_1511), H2228 (RRID: CVCL_1543), H2030 (RRID: CVCL_1517), H358 (RRID: CVCL_1559), and 293T (RRID: CVCL_0063) (passage number = 4) were obtained from the Cell Bank of the Chinese Academy of Sciences. Calu-1 cells were cultured in McCoy’s 5A medium, Beas-2B and 293T in Dulbecco's modified Eagle's medium, and the rest cells in RPMI1640, supplemented with 10% fetal bovine serum (Gibco) and 1% penicillin–streptomycin (Sbjbio). Authentications were obtained by a Human STR Profiling Cell Authentication Service (American Type Culture Collection), and mycoplasma was tested in all cell lines.

To create Sot-resistant H2030/SR cells, H2030 cells were first exposed to 2 nM Sot for 24 h and then cultured without the inhibitor until normal growth resumed. Cells underwent 6 to 10 cycles of drug exposure, followed by doubling the Sot concentration for another 6 to 10 cycles, until the IC_50_ increased more than 10-folds.

To generate a stable cell line, lentivirus-based plasmids pLKO.1 (RRID: Addgene_8453), pLKO.1-shANP were constructed as described previously (10). The lentiviral plasmids used in this study included pU6-Cas9 (P88382, RRID: Addgene_62988), pU6-sgRNA-ANP32A (P65521), pLV-CMV-puro (P80484, RRID: Addgene_123223), pLV-ANP32A (P65538), and pLV-YEATS4-FLAG (P76387), were synthesized by Miaolingbio. Lentiviral plasmids along with packaging plasmids psPAX2 (RRID: Addgene_12260) and pMD2.G (RRID: Addgene_12259) were cotransfected into 293T cells. Viral supernatants were collected 48 h post-transfection and used to infect H2030 and H358 cells with polybrene. Stably transduced cells were selected in puromycin or G418 for 5 days. Full-length details of the plasmids used are provided in the supporting information.

### Xenograft mouse lung cancer model

The Animal Ethics Committee of Jinhua Central Hospital approved the mouse study (license: AL-JHYY2023-39). NOD–SCID mice were obtained from Shanghai Ziyuan Biotechnology. After cell counting, cells were mixed with Matrigel, and a 100 μl suspension containing 1 × 10^7^ cells was injected into the right axillary region of each mouse. Once tumors reached 50 to 100 mm^3^, mice were divided into three groups: control, Sot, and Sot with TSA. TSA was given subcutaneously at 0.5 mg/kg/day, whereas Sot was given orally at 30 mg/kg/day in a 0.5% CMC–Na solution. Mice were weighed daily, and tumor volumes were tracked. Tumor volume = length × (width)^2^/2. On day 30, mice were euthanized, and tumor weights were recorded.

### Online database analysis and verification in lung cancer patient samples

Prognostic and mRNA expression data for lung cancer patients were sourced from The Cancer Genome Atlas database (https://tcga-data.nci.nih.gov/tcga/). Analyses of OS and expression data for ANP32A were conducted *via* the Home for Researchers platform (https://www.home-for-researchers.com/#/). Potential interacting proteins of ANP32A were predicted using the BioGRID database (https://thebiogrid.org/).

To detect ANP32A at the protein and mRNA levels, 66 human lung cancer samples were collected from Jinhua Central Hospital, with histological confirmation and patient consent. The study adhered to *The Declaration of Helsinki* and received ethics approval (no.: AL-JHYY202437). A tissue microarray from Shanghai Outdo Biotech was also used, and two pathologists independently evaluated stained slides. Staining intensity was scored from 1 to 4, and the final score was calculated by multiplying this intensity by the percentage of positive cells. The characteristics of LUAD patients are listed in Table S1.

### Quantitative RT–PCR

Total RNA was extracted utilizing TRIzon Reagent (CW0580S; Cwbio). Complementary DNA synthesis was conducted employing the PrimeScript RT Master Mix (RR036A; Takara). qPCR was subsequently performed using the TB Green Fast qPCR Mix (RR430A; Takara). The primers used for qPCR are detailed in Table S2.

### Immunoprecipitation and Western blotting

For coimmunoprecipitation (IP), cells were lysed on ice for 30 min with IP lysis buffer containing 1% PMSF. After centrifugation, 100 μl of the supernatant was saved as input. The rest was incubated overnight at 4 °C with the FLAG antibody, followed by a 4-h incubation with 20 μl magnetic beads. Beads were washed 3 to -4 times with IP lysis buffer, and proteins were eluted by boiling in loading buffer (LT101; Epizyme) at 95 °C for 10 min and used for Western blotting analysis.

To perform a Western blot, proteins were extracted from lysed cells using radioimmunoprecipitation assay buffer with 1% PMSF. Loading buffer was added to the samples, which were then boiled for 10 min. After cooling, 10 μl of each sample was loaded onto SDS-PAGE gels for electrophoresis. Separated proteins were transferred to polyvinylidene difluoride membranes (IPVH00010; Millipore), blocked with 5% skim milk in Tris-buffered saline with Tween-20 for 1 h, and incubated overnight with primary antibodies at 4 °C. The next day, membranes were washed with Tris-buffered saline with Tween-20, incubated with secondary antibodies for 1 h, and protein bands were visualized using an ECL detection kit (36208ES60; Yeasen Biotechnology) on a Bio-Rad ChemiDoc XRS system. Chemical reagents and antibodies used in cell culture and Western blot, all of which are commercially available products, are listed in Table S3 along with their corresponding RRID identifiers.

### Cell Counting Kit-8 assay

Cells (800/well) were seeded in 96-well plates with 100 μl of culture medium. Viability was measured on days 0 to 6 with the Enhanced Cell Counting Kit-8 (BL1055E; Biosharp). For drug sensitivity tests, cells were seeded at 1500 cells per well under the same conditions and exposed to varying drug concentrations for 24 or 72 h. Viability was measured by Cell Counting Kit-8, and IC_50_ was calculated.

### Cell migration and invasion assay

A total of 2 × 10^4^ cells were suspended in a serum-free medium and placed in the upper chamber of a Transwell apparatus (84052ES12; Yeasen Biotechnology). The lower chamber contained medium with 20% fetal bovine serum. After 24 h, cells migrating into the lower chamber were fixed and stained with crystal violet. For the invasion assay, the upper chamber’s polycarbonate membrane was precoated with Matrigel (356230; Corning) and diluted 1:10 with serum-free medium.

### Apoptosis analysis

Cells (1 × 10^5^/well) were seeded in 6-well plates with 2 ml of culture medium and incubated for 12 h. Cells were treated with different drug concentrations for 24 or 72 h and stained with an Annexin V-PE/7-AAD kit (Vazyme), following the guidelines of the manufacturer. Data were collected by flow cytometry (CytoFLEX; Beckman Coulter), and cell apoptosis was analyzed with FlowJo (version 10.8.1; BD [Becton, Dickinson & Company] Life Sciences).

### 5-Ethynyl-2′-deoxyuridine assay

The BeyoClick EdU-555 Cell Proliferation Detection Kit (C0075S; Beyotime) was used to treat cells (2 × 10^4^) in a 24-well plate with 500 μl of culture medium per well following the manufacturer’s instructions. Microscopic images were acquired with an Olympus BX43 microscope.

### Cell cycle assay

Cells (1 × 10^5^) were seeded into 6-well plates with 2 ml of culture medium and incubated for 12 h. After incubation, cells were collected and stained with the Cell Cycle Detection Kit (KeyGEN BioTECH) following the guidelines of the manufacturer. Data were collected by flow cytometry (CytoFLEX), and the cell cycle was analyzed with ModFit software (Verity Software House).

### H&E staining

H&E Staining Kit (C0105M; Beyotime) was used. Briefly, sections were stained with hematoxylin, followed by a 30-s incubation of eosin solution. Subsequently, sections were dehydrated and mounted using neutral balsam. Microscopic images were acquired with an Olympus BX43 microscope.

### Immunohistochemical staining

Antigen retrieval was performed by utilizing the One-Step Dewaxing/Antigen Retrieval Buffer (pH 6.0) (20×) (Elabscience). The ready-to-use SABC-POD(F) (Rabbit IgG) Kit (catalog no.: SA1028) and the SABC-POD(F) (Mouse IgG) Kit (catalog no.: SA1027) were used (Boster Biological Technology) for the subsequent staining of mouse tumor tissue sections following the manual provided by the manufacturer. Microscopic images were acquired with an Olympus BX43 microscope.

### Chromatin IP assay

Cells were harvested, and ChIP was conducted utilizing the BeyoChIP Enzymatic ChIP Assay Kit (P2083S; Beyotime), following the manufacturer’s protocol. The primers employed for ChIP–qPCR are detailed in Table S2.

### Immunofluorescence assay

A cell climb slip was placed in each well of a 24-well plate. About 2 × 10^4^ cells were seeded and cultured for 12 h. Slides were washed with PBS and fixed with 4% paraformaldehyde for 15 min at room temperature. After washing with PBS containing 3% bovine serum albumin (BSA) (9048-46-8; Yeasen Biotechnology), cells were permeabilized with Immunostaining Strong Permeabilization Buffer (P0097-500 ml; Beyotime) for 20 min at room temperature and washed three times for 5 min each with PBS containing 3% BSA. After blocking with PBS containing 5% BSA for 1 h at room temperature, slides were incubated with primary antibodies overnight at 4 °C, followed by incubation with fluorescent-conjugated secondary antibodies for 2 h at 37 °C in the dark. Coverslips were finally mounted with AntiFade Mounting Medium (with 4',6-diamidino-2-phenylindole) (HY-K1047; MedChemExpress) and stored in a protected environment. Microscopic images were acquired with an NIS microscope (Nikon).

### Transcriptomic sequencing and data analysis

Sot-resistant H2030 cells (H2030/SR) were grown to 70% confluence in 10 cm dishes and treated with 1 μM Sot, 100 nM TSA, or both for 24 h. Cells were harvested, and total RNA was extracted with TRIzon Reagent (CW0580S; Cwbio) for transcriptomic sequencing (Tsingke Biotechnology). The obtained sequencing data were analyzed using the online analysis platform provided by the company (https://cloud.tsingke.com.cn/main/analysisTool). For differential gene expression analysis, genes with |log2 fold change| >1 and *p* < 0.05 were identified as differentially expressed between groups. All identified differentially expressed genes were subsequently subjected to Kyoto Encyclopedia of Genes and Genomes functional enrichment analysis.

### Statistical analysis

For quantification of Western blot analysis, intensity values were obtained using ImageJ (National Institutes of Health). All signals were normalized to the total protein loaded per lane, with histone acetylation levels and phosphorylated Erk further normalized to total histone and total Erk levels, respectively. The data were processed and visualized with GraphPad Prism (version 9.0; GraphPad Software, Inc). Data are presented as the mean ± standard deviation (x ± s). For statistical analysis, an unpaired Student’s *t* test was employed to compare two groups, whereas a one-way ANOVA was conducted for comparisons across multiple groups. Individual data points are overlaid on the bar graphs, with each point representing a biological replicate. A *p* value of less than 0.05 was deemed to indicate statistical significance.

## Data availability

Raw RNA-Seq data have been deposited in the Sequence Read Archive under BioProject accession number PRJNA1337559.

## Supporting information

This article contains supporting information.

## Conflict of interest

The authors declare that they have no conflicts of interest with the contents of this article.
