# Supplementary material for: ANP32A-mediated histone 3 K27 acetylation is essential for sotorasib activity in KRAS-mutant non–small cell lung cancer
Source: J Biol Chem. 2025 Dec 29;302(2):111110. doi: 10.1016/j.jbc.2025.111110 (PMC12857290; doi:10.1016/j.jbc.2025.111110)
Supplement: Supporting Figures and Tables [file mmc1.pdf]

ANP32A-mediated Histone 3 K27 Acetylation is Essential for Sotorasib activity in KRAS-mutant Non-Small Cell Lung Cancer

Kailing Pan[1, 2, 3†], Mingjing Dang[4†], Bo Xu[1], Zan Huang[4*], Xianguo Chen[1, 3, 5*]

[1]Department of Cardiothoracic Surgery, [2]Central Laboratory and Precision Medicine Center, [3]Jinhua Key Laboratory of Cancer Nutrition and Metabolism Research, Jinhua Municipal Central Hospital, Affiliated Jinhua Hospital of Zhejiang University School of Medicine, Jinhua 321000, China.
[4]College of Life Sciences, Hubei Key Laboratory of Cell Homeostasis, Wuhan University, Hubei 430072, China.
[5]College of Mathematical Medicine, Zhejiang Normal University, Jinhua 321000, China.

***Correspondence:**
z-huang@whu.edu.cn (Z.H.); xgchen@zjnu.edu.cn (X.C.)

[†]These authors contributed equally to this work and should be considered co-first authors

List

S-2. Figure S1 ANP32A is tightly associated with lung cancer.

S-3. Figure S2 ANP32A promotes the proliferation of *KRAS*-mutant lung cancer cells.

S-4. Figure S3 ANP32A regulates H3K27 acetylation and interacts with YEATS4.

S-6. Figure S4 ANP32A promotes H3K27Ac by upregulating and interacting with YEATS4.

S-7. Figure S5 Inhibition of histone deacetylation enhances Sotorasib efficacy on KRAS-mutant lung cancer *in vitro* and *in vivo*.

S-8. Figure S6 ANP32A promotes sensitivity to Sotorasib by modulating the p53 signaling pathway via histone acetylation.

S-9. Table S1 Clinical and pathological characteristics of the LUAD patient cohort in the tissue array.

S-12. Table S2 The primers employed for RT-qPCR and ChIP-qPCR.

S-14. Table S3 Chemical reagents and antibodies used in cell culture and Western blot.

S-16. Table S4 Sequences of plasmid inserts.

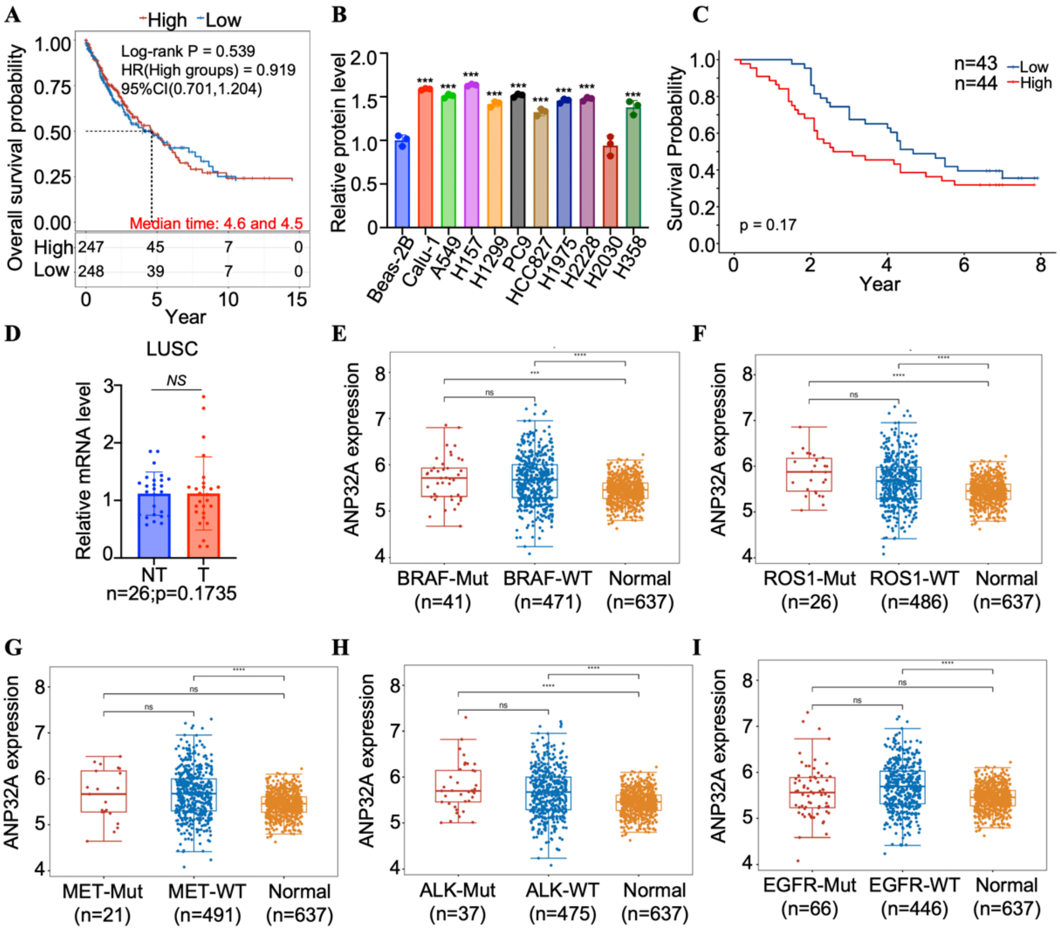

Figure S1 ANP32A is tightly associated with lung cancer. (A) Kaplan-Meier survival analysis on TCGA data shows no correlation of *ANP32A* expression and overall survival rate in LUSC patients. (B) Statistical analysis on ANP32A protein expression in non-transformed Beas-2B cells and various LUAD cell lines (n =3, ***$P < 0.001$). (C) Kaplan-Meier survival analysis shows no correlation of ANP32A protein expression to overall survival rate in LUAD tissue array. (D) No significant difference of *ANP32A* expression in T and NT from our LUSC patient cohort (n=26). (E-I) No significant *ANP32A* expression was observed in LUAD patients with different driver mutations compared to corresponding wild-type LUAD. BRAF (E), ROS1 (F), MET (G), ALK (H), and EGFR (I) mutant LUAD samples were compared to corresponding wild-type LUAD and normal tissues from TCGA. *NS* indicates non-significance.

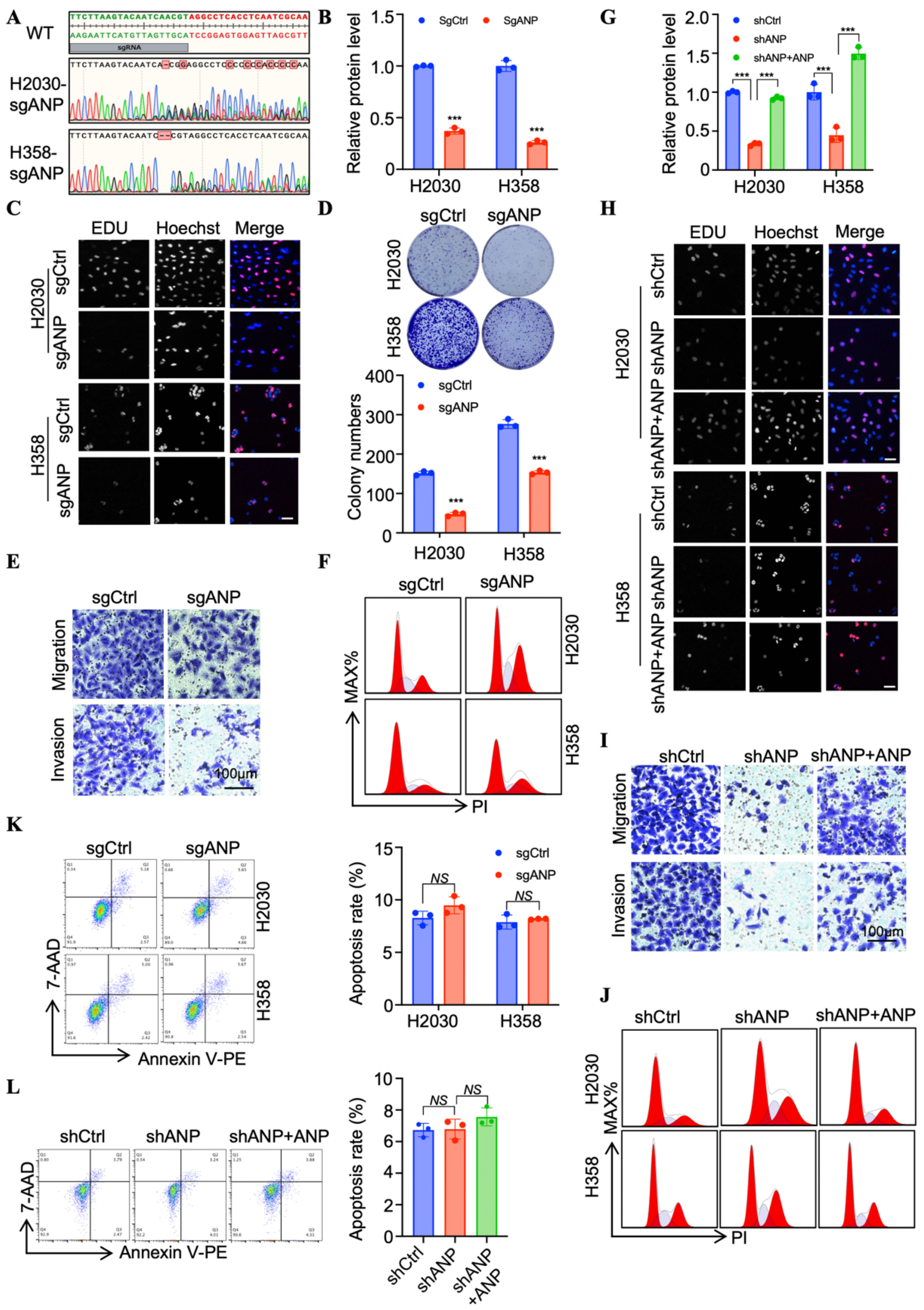

Figure S2 ANP32A promotes the proliferation of *KRAS*-mutant lung cancer cells. (A) Sanger sequencing confirmed that ANP32A was successfully knocked out. (B) The densitometric analysis on ANP32A protein in control (sgCtrl) and knockout (sgANP) H2030 and H358 cells (n =3, ****P < 0.001*). (C) Representative images of EdU assay in control (sgCtrl) and ANP32A-knockout (sgANP) H2030 and H358 cell lines (n=3, ****P < 0.001*). (D) Representative images (top) and statistical analysis (bottom)

of colony formation in sgCtrl and sgANP H2030 and H358 cells (n=3, ****P* < *0.001*). (E-F) Representative images of Transwell migration assay (E) and cell cycle distribution (F) in sgCtrl and sgANP H2030 and H358 cells (n=3). (G) Statistical analysis on ANP32A protein in control (shCtrl), knockdown (shANP), and knockdown with ANP32A reintroduction (shANP+ANP) H2030 and H358 cells (n=3, ****P* < *0.001*). (H-J) Representative images of EdU assay (H), Transwell migration assay (I), and cell cycle distribution (J) in control (shCtrl), ANP32A-knockdown (shANP), and rescue-expressed (shANP+ANP) H2030 and H358 cells (n=3). (K) Representative images (left) and statistical analysis (right) of apoptosis assay in control and ANP32A-knockout H2030 and H358 cell lines (n=3). (L) Representative images (left) and quantitative results (right) of apoptosis in control, ANP32A-knockdown, and rescue-expressed H2030 cells (n=3). * indicates *P* < *0.05*, ** indicates *P* < *0.01*, and *** indicates *P* < *0.001*. *NS* indicates non-significance.

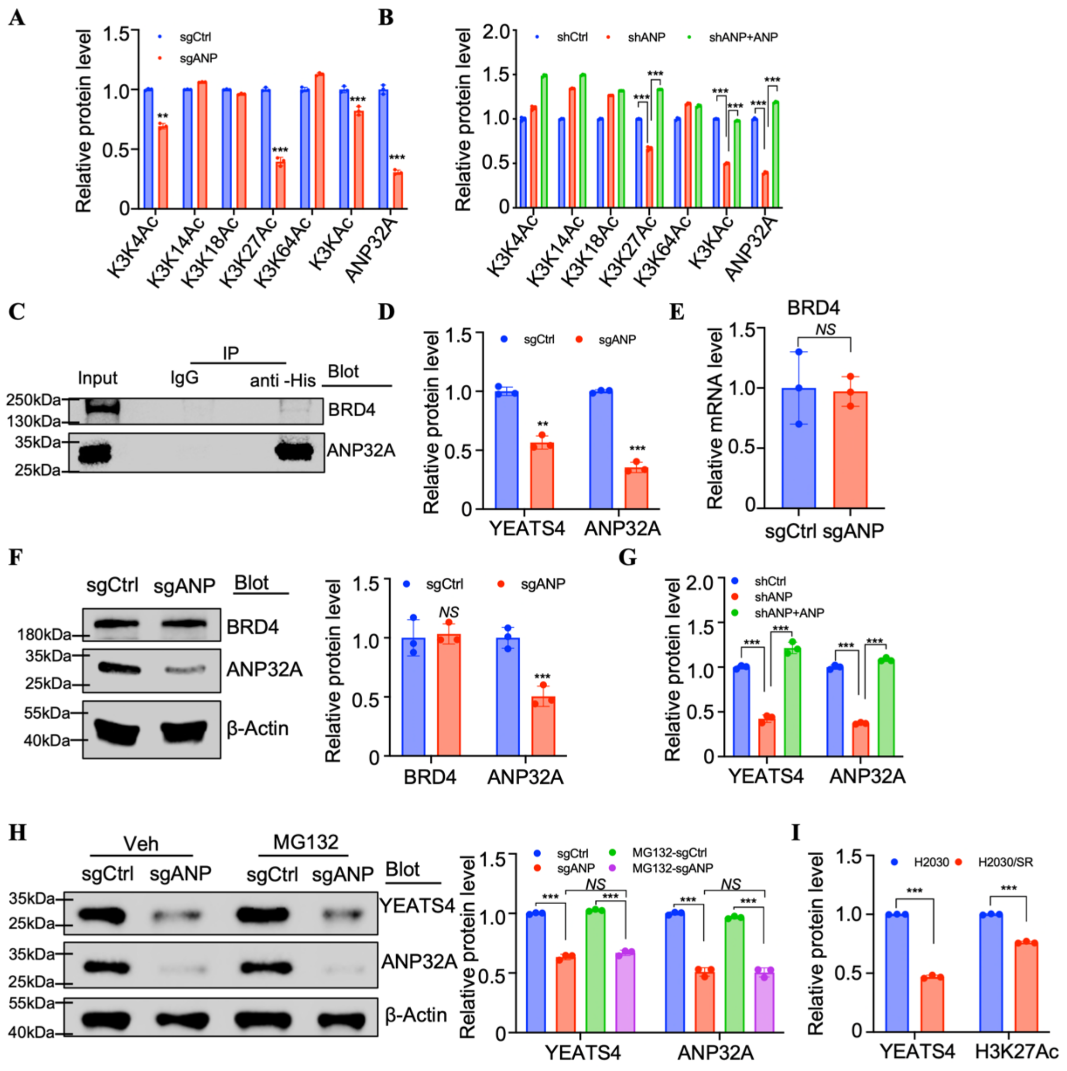

Figure S3 ANP32A regulates H3K27 acetylation and interacts with YEATS4. (A-B) The densitometric analysis showed different histone H3 acetylation levels at various sites in sgCtrl and sgANP H2030 cells (A, n = 3, **$P = 0.009$, ***$P < 0.001$), or in shCtrl, shANP, and shANP+ANP H2030 cells (B, n = 3, ***$P < 0.001$). (C) Co-immunoprecipitation with anti-His antibody using His-ANP32A-overexpressing 293T cells showed no interaction between ANP32A and BRD4. (D) Statistical analyses on YEATS4 protein level in sgCtrl and sgANP H2030 cells (n = 3, **$P = 0.002$, ***$P < 0.001$). (E-F) Analyses on BRD4 mRNA (E) and protein levels (F) in sgCtrl and sgANP H2030 cells (n = 3, ***$P < 0.001$). (G) Statistical analyses evaluated YEATS4 protein levels in shCtrl, shANP, and shANP+ANP H2030 cells (n=3, ***$P < 0.001$). (H) Western blot (left) and statistical analysis (right) of the effect of MG132 on YEATS4 protein (n=3, ***$P < 0.001$). (I) Statistical analysis assessed YEATS4 and H3K27Ac levels in H2030 and H2030/SR cells (n=3, ***$P < 0.001$). * indicates $P < 0.05$, ** indicates $P < 0.01$, and *** indicates $P < 0.001$. *NS* indicates non-significance.

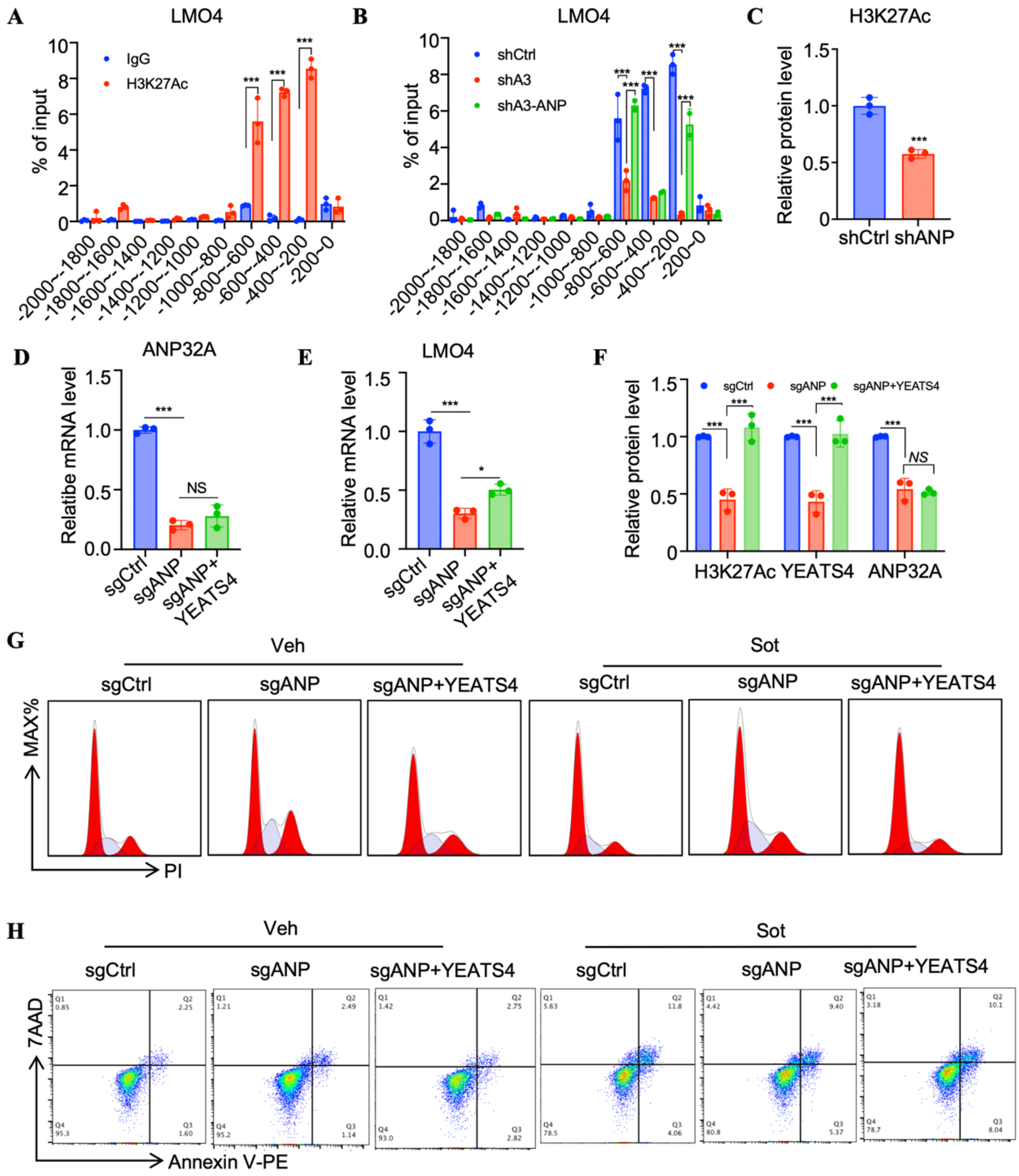

Figure S4 ANP32A promotes H3K27Ac by upregulating and interacting with YEATS4. (A) ChIP-qPCR detection of H3K27Ac levels at different regions of the *LMO4* gene promoter (n=3, ***$P < 0.001$). (B) ChIP-qPCR analysis on H3K27Ac enrichment at various *LMO4* gene promoter regions in shCtrl, shANP, and shANP+ANP H2030 cells (n=3, ***$P < 0.001$). (C) Western blots confirm that ANP32A knockdown impaired the interaction of YEATS4 and H3K27Ac (n=3, ***$P < 0.001$). (D-E) *ANP32A* (D, ***$P < 0.001$) and *LMO4* (E, *$P = 0.025$, ***$P < 0.001$) gene expression in sgCtrl, sgANP, and sgANP+YEATS4 H2030 cells (n = 3). (F) Western blot analyzed ANP32A, YEATS4, and H3K27Ac levels in sgCtrl, sgANP, and sgANP+YEATS4 H2030 cells (n=3, ***$P < 0.001$). (G-H) Representative flow cytometry of apoptosis (G) and cell cycle (H) in shCtrl, shANP, and shANP+YEATS4 H2030 cells

treated with Vehicle or Sotorasib (1 μM) treatment (n=3). * indicates $P < 0.05$, ** indicates $P < 0.01$, *** indicates $P < 0.001$. *NS* indicates non-significance.

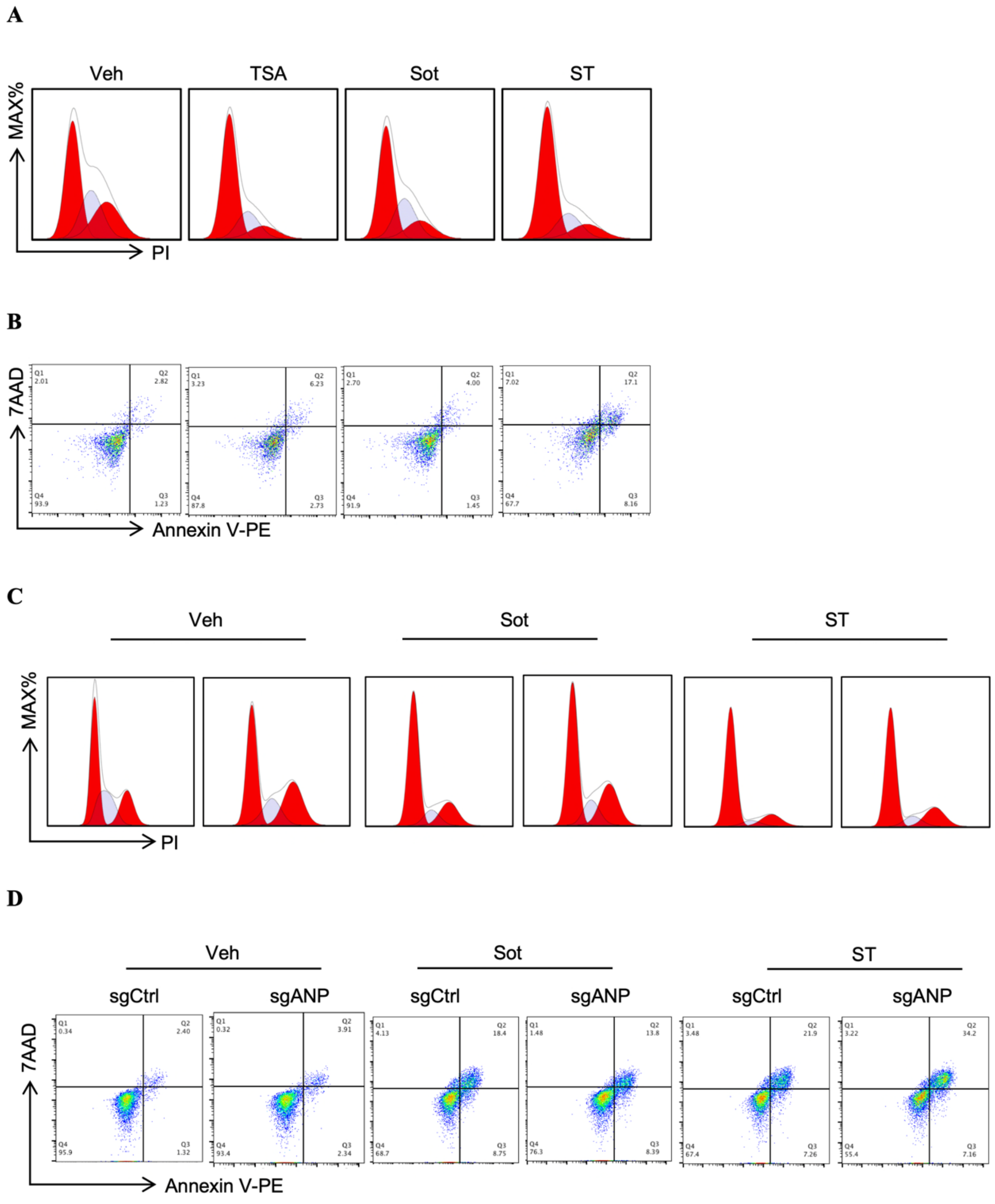

Figure S5 Inhibition of histone deacetylation enhances Sotorasib efficacy on KRAS-mutant lung cancer *in vitro* and *in vivo*. (A–B) Representative flow cytometry of cell cycle (A) and apoptosis (B) in H2030/SR cells treated with Vehicle, TSA (100 nM), Sotorasib (Sot, 1 μM), or the combination of TSA and Sotorasib (ST) (n=3). (C) Representative flow cytometry of cell cycle in sgCtrl and sgANP cells treated with Vehicle (Veh), Sot, (1 μM), or ST (Sot plus TSA; Sot, 1 μM; TSA 100 nM) (n=3). (D)

Representative flow cytometry of apoptosis in sgCtrl and sgANP cells treated with Veh, Sot (10 μM), or ST (Sot plus TSA; Sot, 10 μM; TSA 100 nM) (n=3).

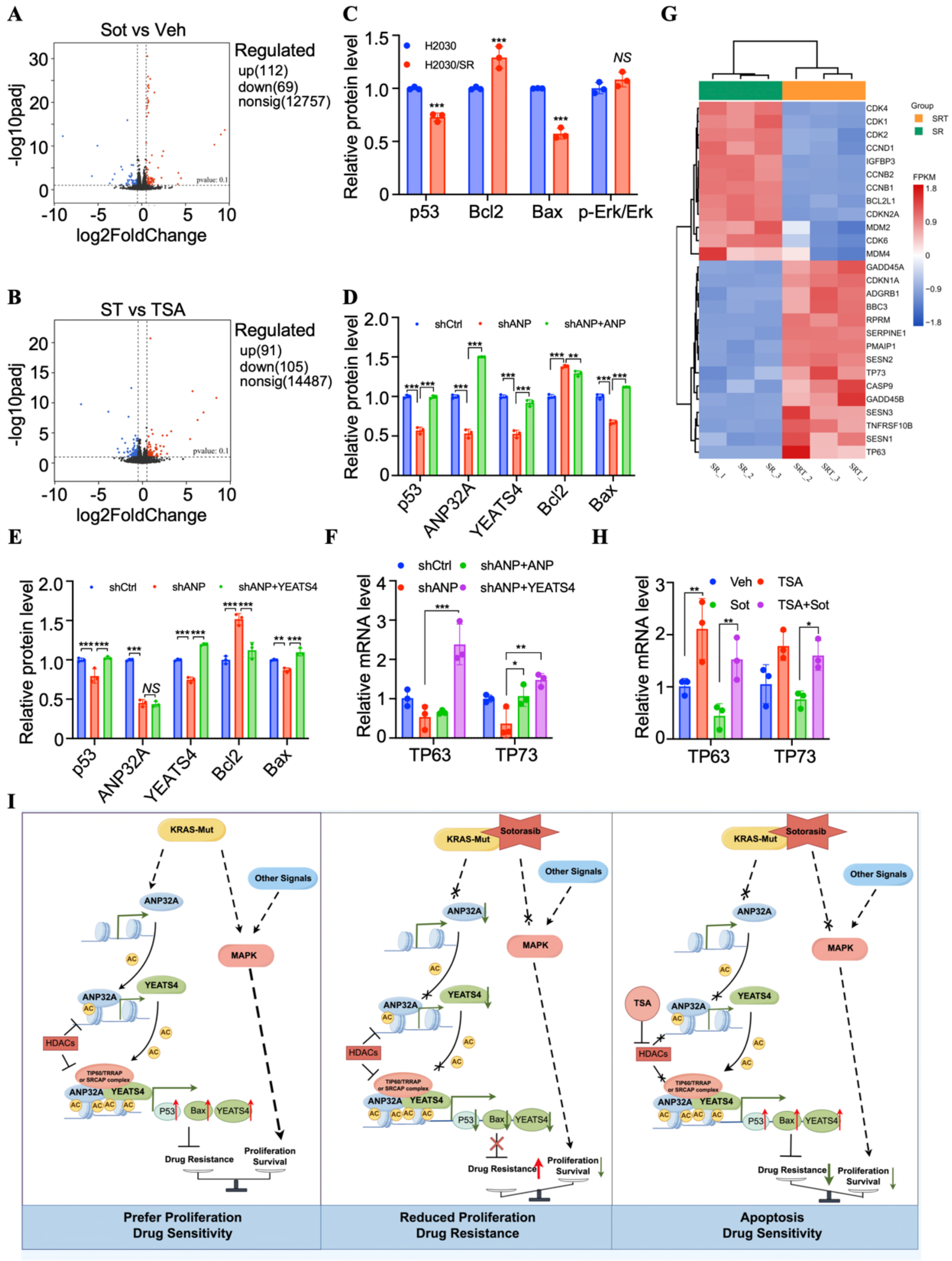

Figure S6 ANP32A promotes sensitivity to Sotorasib by modulating the p53 signaling pathway via histone acetylation. (A-B) Volcano plots show differentially expressed genes in H2030/SR cells treated with Veh, Sot (1 μM), TSA (100 nM), or ST for RNA-Seq analysis. Sot-treated group is compared to

Veh-treated group in (A), and TSA-treated group is compared to ST-treated group in (B). (C) Western blot analysis assessed p53 and MAPK signaling molecules in H2030 and H2030/SR cells (n = 3, ***$P < 0.001$). (D) Statistical analysis on the levels of p53 signaling molecules in shCtr, shANP and shANP-ANP cells (n = 3, **P = 0.004, ***P < 0.001). (E) Western blot analysis assessed p53 signaling molecules in shCtrl, shANP and shANP-YEATS4 cells (n=3, **$P = 0.007$, ***$P < 0.001$). (F) The mRNA levels of *TP63* and *TP73* in shCtrl, shANP, shANP+ANP and shANP+YEATS4 cells (n=3, *$P = 0.038$, **$P = 0.001$, ***$P < 0.001$). (G) Heatmap showing expression of p53 pathway genes in Sot-treated versus ST-treated groups. (H) The mRNA expression of *TP63* and *TP73* was assessed at mRNA levels in H2030/SR cells treated with Veh, Sot (1 μM), TSA (100 nM), or ST (n=3, *$P = 0.045$, **$P = 0.006$ *vs. Veh, 0.007 vs. Sot*). (I) The graphic diagram * indicates $P < 0.05$, ** indicates $P < 0.01$, *** indicates $P < 0.001$. *NS* indicates non-significance.

Table S1. Clinical and pathological characteristics of the LUAD patient cohort in the tissue array.

ANP32A level	WHO grade	Death	Survival month	Gender	Age	T	N	M
0	1	1	66	0	58	2	0	0
0	2	1	24	1	62	1	1	0
0	2	0	95	1	49	2	1	0
1	3	1	2	0	64	1	2	0
0	1	0	95	0	61	1	0	0
1	1	1	52	0	63	1	0	0
1	2	0	94	1	67	2	0	0
0	1	0	94	0	62	1	0	0
0	1	1	84	0	62	1	0	0
0	3	1	24	1	72	3	1	0
0	2	0	90	0	53	1	1	0
0	2	0	89	0	48	1	1	0
0	1	0	89	1	59	1	0	0
0	1	0	89	1	67	2	0	0
0	2	1	27	1	71	2	1	0
1	2	1	18	1	58	2	0	0
1	3	1	26	0	44	1	2	0
1	1	1	37	0	68	2	0	0
0	1	1	70	0	66	1	0	0
0	2	1	48	1	50	1	1	0
0	1	1	52	0	66	1	0	0
1	1	1	60	0	48	1	0	0
0	1	1	29	1	60	2	0	0
0	1	0	84	0	62	1	0	0
1	1	0	84	1	46	1	0	0
0	3	1	24	1	69	2	2	0
1	2	1	14	1	67	1	1	0
0	3	1	22	0	54	2	2	0
0	1	1	24	1	72	1	0	0
0	1	1	63	0	65	1	0	0
1	3	1	26	1	74	4	0	0
1	1	0	84	1	70	1	0	0
1	3	0	84	1	47	1	2	0
0	1	0	84	1	61	1	0	0
1	2	1	52	0	66	2	0	0
1	3	1	25	1	62	2	2	0
1	2	0	84	1	37	1	1	0
0	1	1	36	0	66	1	0	0
0	1	1	36	1	44	1	0	0
1	2	1	25	0	42	2	1	0
1	1	1	7	1	57	1	0	0
0	1	0	83	1	67	1	0	0

0	2	0	83	0	72	2	0	0
1	1	1	19	1	49	2	0	0
1	1	1	31	0	67	2	0	0
0	3	1	36	0	46	2	3	0
1	1	1	41	1	53	1	0	0
1	3	0	83	1	47	3	2	0
1	2	0	83	1	65	2	0	0
1	1	0	83	0	57	1	0	0
1	3	1	5	1	65	2	3	0
1	2	1	7	1	70	2	1	0
1	3	1	30	1	75	4	0	0
0	2	1	25	1	68	1	1	0
1	2	1	20	1	49	3	0	0
1	3	1	25	1	58	3	1	0
0	2	1	30	1	48	2	0	0
1	3	0	83	0	51	2	2	0
0	1	1	18	1	51	1	0	0
0	1	1	51	0	51	1	0	0
1	3	1	17	0	58	2	2	0
0	1	1	66	1	67	1	0	0
0	1	1	51	0	60	1	0	0
1	1	1	69	0	68	1	0	0
1	1	0	82	1	50	1	0	0
1	1	0	82	0	53	1	0	0
0	1	0	82	1	62	1	0	0
0	2	0	82	1	59	1	1	0
1	3	1	13	0	64	2	2	0
1	1	1	50	1	60	1	0	0
0	2	1	41	1	53	2	1	0
1	2	1	22	0	68	3	0	0
0	3	1	25	0	40	2	2	0
1	2	1	28	0	56	2	1	0
1	1	0	80	1	59	1	0	0
0	1	0	80	0	60	1	0	0
1	2	1	17	1	79	2	1	0
1	3	0	80	0	72	2	2	0
0	1	1	56	1	61	1	0	0
1	3	1	17	1	70	3	2	0
1	1	1	65	0	57	1	0	0
0	1	0	79	0	59	1	0	0
0	1	0	79	1	50	1	0	0
1	2	0	77	0	50	1	1	0
0	1	1	49	0	69	1	0	0
0	1	1	52	0	60	1	0	0

1	2	1	11	0	65	3	0	0

Table S1. Clinical and pathological characteristics of the LUAD patient cohort in the tissue array. Values in the ANP32A column represent expression levels (0, low; 1, high). WHO grade is indicated as 1, 2, or 3. Vital status is coded as 0 (alive) or 1 (deceased). The Survivalmonth column indicates the overall survival time in months. Gender is defined as 0 (female) or 1 (male). Age refers to the age of patient at the time of surgery. The T, N, and M columns denote the pathological TNM stage.

Table S2 The primers employed for RT-qPCR and ChIP-qPCR.

	**Sequence (5'->3')**
**RT-qPCR**	
ANP32A-F	AGCCTTCAAAGTCCTAAAACGC
ANP32A-R	CCAGGACAAGTTCTTTCACATCAG
TP53-F	ACAGCTTTGAGGTGCGTGT
TP53-R	TTGGGCAGTGCTCGCTTA
DR5-F	CCAAGACCCTTGTGCTCGTT
DR5-R	CTTCTGAGATATGGTGTCCAGGT
TP63-F	TGAAACTTCACGGTGTGCCA
TP63-R	AATTCAGTGCCAACCTGGGG
TP73-F	CCAGATCCATGCCTCGTCC
TP73-R	GGAGCAGACTGTCCTTCGTT
GADD45A-F	AGAGCAGAAGACCGAAAGGATG
GADD45A-R	CTTCGTACACCCCGACAGTG
YEATS4-F	GAGAATGGCCGAATTTGGGC
YEATS4-R	CCGAGCAACATTACCGTAAACT
LMO4-F	CCAGACCAGAAGGTCTGCTA
LMO4-R	CGGGAAGGGCAATCAAGACT
GAPDH-F	ATGGGGAAGGTGAAGGTCG
GAPDH-R	CTCCACGACGTACTCAGCG
**ChIP-qPCR**	
YEATS4-1-F	GGATGAGTCAAGGTAGCGGAG
YEATS4-1-R	TCAGCCTGTGTTGTTGGGTT
YEATS4-2-F	ACGCCCCCAAACCAAATTTC
YEATS4-2-R	GGATGTCACTGCATTTCAACCA
YEATS4-3-F	CACTAATATAACTAAGCCCTGAGC
YEATS4-3-R	AGTAATTCCACTTAACAACTGT
YEATS4-4-F	CTCCTCCATCCCCATCAAGC
YEATS4-4-R	AAACTGAGTATTGATCCCCC
YEATS4-5-F	CACCACGCTCGGCTAATTTT

YEATS4-5-R	CGAGGTCAGGAGATCGAGAC
YEATS4-6-F	GCCCGGCCAATACTCAGTAT
YEATS4-6-R	GGTACACAGAAAACTAAGCGGC
YEATS4-7-F	TCTCGGTCAAATCTCCTCTTGA
YEATS4-7-R	TCTTTGGGAGACACTGCTTC
YEATS4-8-F	TCCCTCATTTTGGTGGATCAGA
YEATS4-8-R	ACTCATCCAACTACCAATCTAGTGT
YEATS4-9-F	CCTCAGAGTTTTGAAGACAGTGC
YEATS4-9-R	GGAAAAAGAGATCCCACACCCT
YEATS4-10-F	ACTCATCTCCTTAACATCTGGACA
YEATS4-10-R	GCAGTCACATCACAGGACCA
LMO4-1-F	TCATGCCCATCTGTAGCTGT
LMO4-1-R	TGATGAATTCACCTTGTGCACA
LMO4-2-F	AATGCCCTCTACCTGACTGT
LMO4-2-R	ATCTGGCGAGACAATGCAGA
LMO4-3-F	CCCACAAGGCTGGTTTTACTTA
LMO4-3-R	GGCCTGATTATTGTTAGTAAGTTCCA
LMO4-4-F	TCGGCCTTGGAAAAAGACTCT
LMO4-4-R	TTTGCAATCTCACAGCCTCT
LMO4-5-F	GCTCCCTACATCACCAGCAA
LMO4-5-R	TACTGCCTACTTAGCCTCCC
LMO4-6-F	TGTTTCTACCTTGTTAACTAAGCTTCC
LMO4-6-R	CGCACAAGCACACAGAAGAG
LMO4-7-F	TCTCAGATAGAGGGGATGGCA
LMO4-7-R	ATCAGGTGGGGTGAAGTGAC
LMO4-8-F	CTTCCCTTCTCCTCTGTCGG
LMO4-8-R	TAAAACCTGCCAATCCGGGG
LMO4-9-F	ATTCCGCCTGAACAATCCGG
LMO4-9-R	CGCGGCTCTTACACATGTGT
LMO4-10-F	GGGCTGTTGTGTCTGCGA
LMO4-10-R	TCACTTTGTATATTCCCCCGC
TP53-1-F	CGGATTACTTGCCCTTACTT
TP53-1-R	CTCAAAACTTTTAGCGCCAG
TP53-2-F	ACGGAAAGCCTTCCTAAAAA
TP53-2-R	TATGAAGGGTGGAAGGAAGA
TP53-3-F	CAGCACTGATATAGGCACTC
TP53-3-R	AGTACATGGAAACGTAAGCC
TP53-4-F	TGAACGCTTCTATCTTGGC
TP53-4-R	GCAGGTGCTTTAAGAATTACC
TP53-5-F	CATCCCGGATCAGATTTCG
TP53-5-R	AACGGGAAACCTTCTAACCT
TP53-6-F	TAACGGTTGAGTCTCCAAAG
TP53-6-R	ACAGGTGGGTTTCTTTAGC

TP53-7-F	GGAGTGGAGAGAGAAACTGG
TP53-7-R	CGCTTCCCCGATGAATAAAA
TP53-8-F	TTCTTCTTCAGAAAGGCTCC
TP53-8-R	AATCGAGGAGCAAGAACTTT
TP53-9-F	GGGACAATAAACCTGGGTC
TP53-9-R	GAAGTGTGAGGTCGATCTGT
TP53-10-F	TTACAGTGAAAATCTCGGGG
TP53-10-R	AGAACTTCTTGAAAGGCTGT

Table S2 The primers employed for RT-qPCR and ChIP-qPCR. F, forward primer; R, reverse primer. ChIP-qPCR primers 1-10 amplify sequential 200-bp genomic regions upstream of the transcription start site, specifically amplicons located at -2000 to -1800, -1800 to -1600, -1600 to -1400, -1400 to -1200, -1200 to -1000, -1000 to -800, -800 to -600, -600 to -400, -400 to -200, and -200 to 0.

Table S3. Chemical reagents and antibodies used in cell culture and Western blot.

Antibodies	Company (Denotations, RRID)	Dilutions
Anti-ANP32A	Proteintech Cat# 15810-1-AP, RRID: AB_2056302	1:1000
Anti-β-actin	Proteintech Cat# HRP-66009, RRID:AB_2883836	1:5000
Anti-Histone3	Huabio Cat# M1309-1, RRID:AB_3073064	1:10000
Anti-H3Kac	Active Motif Cat# 61637, RRID:AB_2793714	1:1000
Anti-H3K4Ac	ABclonal Cat# A17019, RRID:AB_2768207	1:1000
Anti-H3K14Ac	ABclonal Cat# A7254, RRID:AB_2737401	1:1000
Anti-H3K18Ac	ABclonal Cat# A7257, RRID:AB_2767801	1:1000
Anti-H3K27Ac	Proteintech Cat# 82902-1-RR, RRID:AB_3670631	1:1000
Anti-H3K64Ac	Thermo Fisher Scientific Cat# PA5-121384, RRID:AB_2914956	1:1000
Anti-YEATS4	Proteintech Cat# 11050-1-AP, RRID:AB_2217331	1:1000
Anti-BRD4	Proteintech Cat# 28486-1-AP, RRID:AB_2918170	1:1000
Anti-ANP32A	Proteintech Cat# 67687-1-Ig, RRID:AB_2882880	1:50
Anti-Ki67	Proteintech Cat# 27309-1-AP, RRID:AB_2756525	1:100
Anti-BaX	Huabio Cat# ET1603-34, RRID:AB_3069679	1:1000
Anti-Bcl2	Huabio Cat# HA721235, RRID:AB_3072355	1:1000
Anti-p53	Huabio Cat# ET1601-13, RRID:AB_3069595	1:1000
Anti-CCD1	Huabio Cat# ET1601-31, RRID:AB_3069614	1:1000
Anti-ERK1/2	Huabio Cat# ET1601-29, RRID:AB_3069611	1:1000
Anti-perk1/2	Huabio Cat# ET1610-13, RRID:AB_3069896	1:1000
Anti-flag	Proteintech Cat# 20543-1-AP, RRID:AB_11232216	1:1000
HRP-conjugated Goat Anti-Rabbit IgG(H+L)	Proteintech Cat# SA00001-2, RRID:AB_2722564	1：5000
HRP-conjugated Goat Anti-Mouse IgG(H+L)	Proteintech Cat# SA00001-1, RRID:AB_2722565	1：5000
CoraLite488-conjugated Goat Anti-Rabbit IgG(H+L)	Proteintech Cat# SA00013-2, RRID:AB_2797132	1:500
CoraLite594 –conjugated Goat Anti-Mouse IgG(H+L)	Proteintech Cat# SA00013-3, RRID:AB_2797133	1:250
Trichostatin A	MCE (HY-15144)	
Sotorasib	MCE (HY-114277)	

Table S4. Sequences of plasmid inserts.

Type	Sequence
sgRNA	5’-TTCTTAAGTACAATCAACGT-3’
shRNA	5’-CCTGAAGATGAGGGAGAAGAtctcgagaTCTTCTCCCTCATCTTCAGG-3’
ANP32A	5’-<br>GCCACCatggagatgggcagacggattcatttagagctgcggaacaggacgccctctgatgtgaaagaacttgtcctg<br>gacaacagtcggtcgaatgaaggcaaactcgaaggcctcacagatgaatttgaagaactggaattcttaagtacaatcaac<br>gtaggcctcacctcaatcgcaaacttaccaaagttaaacaaacttaagaagcttgaactaagcgataacagagtctcagggg<br>gcctggaagtattggcagaaaagtgtccgaacctcacgcatctaaatttaagtggcaacaaaattaaagacctcagcacaat<br>agagccactgaaaaagttagaaaacctcaagagcttagaccttttcaattgcgaggtaaccaacctgaacgactaccgaga<br>aaatgtgttcaagctcctcccgcaactcacatatctcgacggctatgaccgggacgacaaggaggcccctgactcggatgc<br>tgagggctacgtggagggcctggatgatgaggaggaggatgaggatgaggaggagtatgatgaagatgctcaggtagtg<br>gaagacgaggaggacgaggatgaggaggaggaaggtgaagaggaggacgtgagtggagaggaggaggaggatgaa<br>gaaggttataacgatggagaggtagatgacgaggaagatgaagaagagcttggtgaagaagaaaggggtcagaagcga<br>aaacgagaacctgaagatgagggagaagatgatgaccatcatcatcatcatcactaa-3’
YEATS4	5’-<br>GCCACCatgttcaagagaatggccgaatttgggcctgactccggcgggagagtaaagggtgttactatcgttaaacca<br>atagtttacggtaatgttgctcggtattttggaaagaaaagagaagaagatgggcacactcatcagtggacagtatatgtgaa<br>accatatagaaatgaggatatgtcagcatatgtgaagaaaatccagtttaaattacatgaaagctatggcaatcctttaagagtt<br>gttactaaacctccatatgaaattactgaaacaggatggggtgaattcgaaataatcatcaaaatatttttcattgaccctaatga<br>aagacctgtaaccctgtatcatttgctaaagctgtttcaatcagacaccaatgcaatgctggggaaaaagacagtggtttcag<br>agttctatgatgaaatgatatttcaagacccaacagcaatgatgcaacaattattgacaacatctcgtcagctaacattaggag<br>cctataagcatgaaacagaatttgcagagcttgaagtgaaaaccagagaaaaattagaagctgctaagaaaaaaacaagct<br>ttgagattgcagagcttaaggagagattaaaagcaagtcgtgaaactataaattgtttaaaaaatgaaatcagaaaacttgaa<br>gaagatgaccaagcaaaagacataggatccGACTACAAAGACCATGACGGTGATTATAAAG<br>ATCATGACATCGACTACAAGGATGACGATGACAAGTA-3’
